# Structural basis of asymmetric DNA methylation and ATP-triggered long-range diffusion by EcoP15I

**DOI:** 10.1038/ncomms8363

**Published:** 2015-06-12

**Authors:** Yogesh K. Gupta, Siu-Hong Chan, Shuang-yong Xu, Aneel K. Aggarwal

**Affiliations:** 1 Department of Structural and Chemical Biology, Icahn School of Medicine at Mount Sinai, Box 1677, 1425 Madison Avenue, New York, New York 10029, USA; 2 New England Biolabs Inc., 240 County Road, Ipswich, Massachusetts 01938, USA

## Abstract

Type III R–M enzymes were identified >40 years ago and yet there is no structural information on these multisubunit enzymes. Here we report the structure of a Type III R–M system, consisting of the entire EcoP15I complex (Mod_2_Res_1_) bound to DNA. The structure suggests how ATP hydrolysis is coupled to long-range diffusion of a helicase on DNA, and how a dimeric methyltransferase functions to methylate only one of the two DNA strands. We show that the EcoP15I motor domains are specifically adapted to bind double-stranded DNA and to facilitate DNA sliding via a novel ‘Pin' domain. We also uncover unexpected ‘division of labour', where one Mod subunit recognizes DNA, while the other Mod subunit methylates the target adenine—a mechanism that may extend to adenine N6 RNA methylation in mammalian cells. Together the structure sheds new light on the mechanisms of both helicases and methyltransferases in DNA and RNA metabolism.

## Abstract (web summary)

Type III restriction–modification enzymes consists of two methylation and one or two restriction subunits. Here the authors report the structure of the full EcoP15I complex bound to DNA, which suggests mechanisms for ATP hydrolysis dependent diffusion along DNA and how a dimeric methyltransferase modifies only one DNA strand.

## Graphical abstract

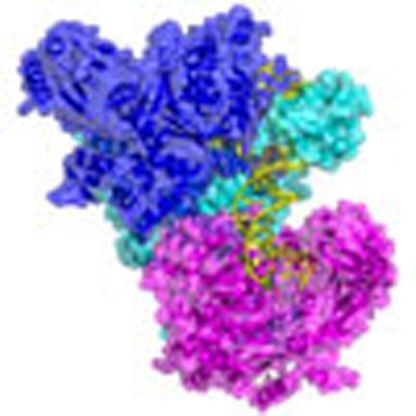

We provide here a basis for two prevailing questions in the study of DNA helicases and DNA methyltransferases. First, how is ATP hydrolysis coupled to long-range diffusion of a helicase on DNA? And second, how does a dimeric methyltransferase methylate one DNA strand but not the other? Both of these activities are embodied in the Type III restriction–modification (R–M) systems in bacteria and archaea, which play roles analogous to the innate immune system in higher eukaryotes^1^,^2^,^3^,^4^. R–M systems are categorized in four groups (Type I–IV) based on their subunit assembly, cofactor requirements and associated cleavage patterns^5^.

EcoP15I is a prototype of the Type III R–M family and consists of two methylation (Mod) and one (or two) restriction (Res) subunits^6^, resulting in a Mod_2_Res_1_ or Mod_2_Res_2_ complex^7^,^8^,^9^,^10^. The Mod subunits are responsible for DNA recognition and methylation, while the Res subunits are responsible for ATP hydrolysis and cleavage. Cleavage only occurs if the two recognition sites (CAGCAG) are in an inverted-repeat orientation, arranged either as ‘head-to-head' or ‘tail-to-tail'^11^,^12^. The sites can be separated by thousands of base pairs but ATP hydrolysis is absolutely required for cleavage^8^.

According to classical helicase mechanisms, motion of EcoP15I should require one ATP per base pair moved on the DNA. However, even though EcoP15I contains the standard helicase motifs found in members of superfamily 2 (SF2) helicases and translocases^13^,^14^, the enzyme communicates over thousands of base pairs by consuming only a few ATPs^15^,^16^. A number of competing models have been proposed for this long-range communication, including classical translocation and three-dimensional DNA looping^17^,^18^. More recently, single-molecule and ensemble fluorescence measurements have shown that EcoP15I undergoes random one-dimensional diffusion along the DNA^16^,^19^. The diffusion coefficient is one of the largest yet measured for a DNA sliding process (*D*=0.92±0.06 μm^2^ s^−1^)^20^, with DNA cleavage only occurring when the freely diffusive EcoP15I collides with a stationary enzyme bound to the second DNA site^19^ (Supplementary Fig. 1). Together, these results posit a new functionality for helicases, as molecular switches for long-lived DNA sliding (rather than conventional DNA/RNA unwinding or stepwise translocation)^21^. EcoP15I hydrolyses ∼30 ATP molecules in two steps (a fast consumption of ∼10 ATP molecules followed by a slower consumption of ∼20 ATP molecules), which switches the enzyme into a distinct structural state that can diffuse on DNA over long distances^19^. A similar sliding-based mechanism has been proposed for the mismatch repair protein MutS (and its eukaryotic homologue), where nucleotide exchange (rather than hydrolysis) triggers sliding on DNA after mismatch recognition^22^,^23^,^24^. Altogether, ATP-triggered sliding is an emerging theme in helicase-like enzymes, but questions about the mechanism remain unanswered due to the lack of structural data.

Intriguingly, the EcoP15I Mod subunit is also unusual in that it functions as a dimer^25^ as compared to monomeric methyltransferases^26^,^27^. In this respect, it is similar to several DNA methyltransferases in mammals (Dnmt3a/3b/3L) and plants (Domains rearranged methyltransferase 2, DRM2) that also function as dimers or other higher order oligomers in processes ranging from *de novo* DNA methylation to RNA-directed DNA methylation^28^,^29^. Similar to DRM2, for example, EcoP15I methylates one DNA strand but not the other. The EcoP15I mode of action also extends to the methylation of adenine N6 in mammalian mRNAs, mediated by a heterodimer of methyltransferase-like 3 and 14 (METTL3 and METTL14), which has been shown to be critical for cellular homeostasis and stem cell commitment and differentiation^30^,^31^. Like the Mod subunit, METTL3 and METTL14 belong to the β class of amino methyltransferases and may operate in a similar dimer mode as EcoP15I. Despite the availability of several crystal structures of dimeric DNA methyltransferases (all in DNA-free form), the structural basis for asymmetric DNA (and RNA) methylation has remained mysterious.

Although Type III R–M enzymes were identified >40 years ago^1^,^2^,^3^,^4^,^32^, there is still no structural information on these multisubunit enzymes that encompass DNA methylation, DNA translocation and DNA cleavage activities all within the same complex. We report here the structure of the entire EcoP15I complex (Mod_2_Res_1_) bound to its DNA substrate. The structure is only the second (after *S. solfataricus* RAD54) of an SF2 helicase with a duplex DNA bound to the motor domains^33^. As such, it provides new insights into the mechanisms of DNA translocation and the nature of the conformational change that switches EcoP15I into a long-lived sliding machine. The structure is also the first, to our knowledge, of a dimeric DNA methyltransferase bound to DNA. It reveals a remarkable division of labour, where one Mod subunit recognizes the DNA, while the other Mod subunit methylates the target adenine base. Together these structural features shed new light on the diversity of helicases and methyltransferases in DNA and RNA metabolism.

## Results

### Structure determination

The EcoP15I holoenzyme was co-crystallized with a 20-mer DNA duplex containing a single EcoP15I recognition site (CAGCAG). The best co-crystals were obtained in the presence of AMP and diffracted to ∼2.6 Å resolution with synchrotron radiation. The co-crystals belong to space group P4_1_2_1_2 with unit cell dimensions of *a*=*b*=101 Å, *c*=533 Å and *α*=*β*=*γ*=90^o^ and contain one EcoP15I/DNA/AMP complex in the crystallographic asymmetric unit (Table 1). The structure was determined by the multiple isomorphous replacement with anomalous scattering (MIRAS) method and refined to 2.6 Å resolution (Table 1). The refined model consists of two Mod subunits (ModA, residues 13–644; ModB, residues 2–644), one Res subunit (residues 7–810), 20-mer DNA (nucleotides 1–20 on each strand), one AMP molecule, 3 ions and 103 solvent molecules. Regions of protein with no electron density were omitted, and amino acids with weak side chain densities were modelled as alanines (Supplementary Table 1). The current model lacks the endonuclease portion of the Res subunit due to the lack of electron density for this region.

### Overall architecture

**Figure 1:**
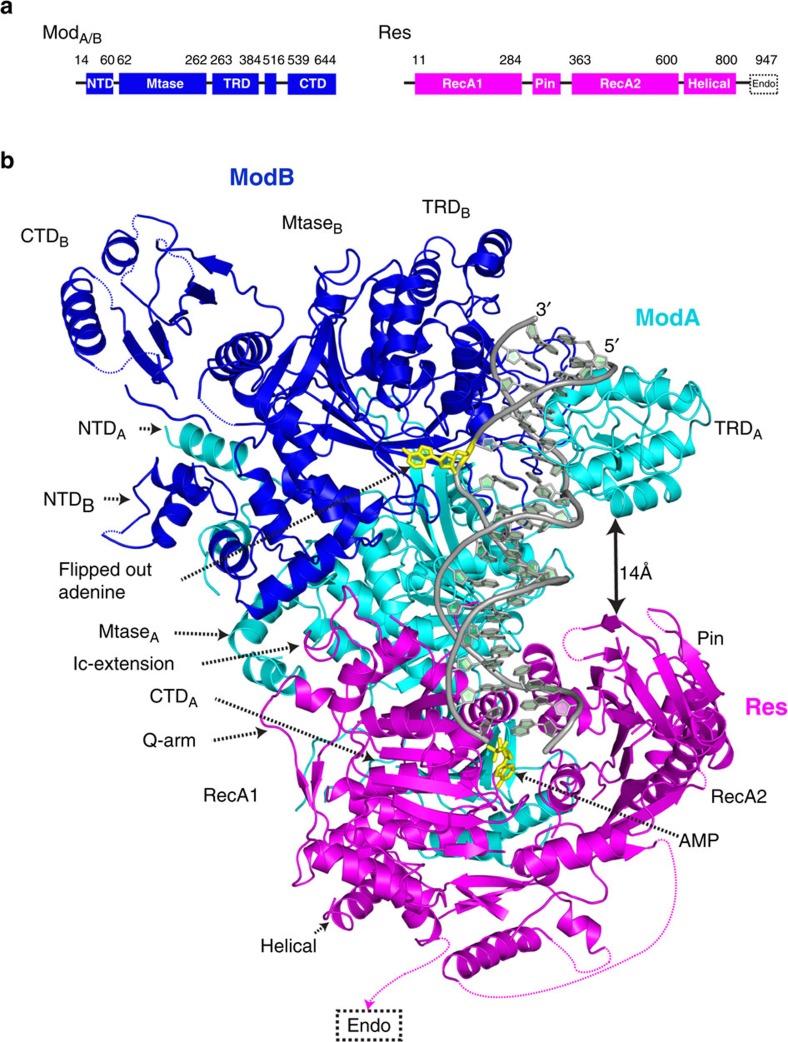
Overall structure of EcoP15I/DNA/AMP complex. (**a**) The domain arrangements of Mod and Res subunits. (**b**) An overall view of the Mod and Res subunits (Mod_2_Res_1_) bound to DNA and AMP. The two Mod protomers, ModA and ModB are shown in cyan and blue, respectively, whereas the Res subunit is shown in magenta. The DNA is shown in grey, with the exception of the extrahelical adenine base (yellow). The AMP molecule is shown in yellow. ModA recognizes DNA through base-specific interaction from its TRD (TRD_A_) and interacts with Res through its MTase domain and CTD. The TRD of ModB (TRD_B_) does not enter the DNA major groove. CTD of ModA (CTD_A_) interacts with the Res subunit, whereas the CTD of ModB (CTD_B_) is exposed to solvent. ModA and ModB dimerize via their NTDs (NTD_A/B_) and central MTase domains (MTase_A/B_). AMP binds in a cleft between the RecA1 and RecA2 motor domains of the Res subunit. The endonuclease domain that ensues the helical spacer is disordered and labelled in a dashed box. The proximity of TRD_A_ of ModA and Pin domain of Res (interdomain distance ∼14 Å) is highlighted by a double-headed arrow. The intervening loops in the structure that are not modeled due to weak density are represented by coloured dashes.

The EcoP15I Mod_2_Res_1_ heterotrimer embraces the DNA duplex and makes extensive protein–DNA contacts. The Mod subunits engage the upstream portion of the DNA duplex that contains the CAGCAG recognition sequence (Fig. 1). The target adenine (CAGC**A**G) rotates out of the DNA helix and enters the catalytic pocket of one of the two Mods, ModB. The other Mod, ModA, makes the majority of base-specific contacts with the CAGCAG recognition sequence. The Res subunit interacts with the downstream portion of the DNA, approximately one half-turn away from recognition site (Fig. 1). Only the helicase core of the Res subunit is visible in the electron density map; the cleavage domain is disordered and may only become ordered when the enzyme collides with another EcoP15I complex and becomes cleavage competent. AMP lies in a cleft in the helicase core.

**Figure 2:**
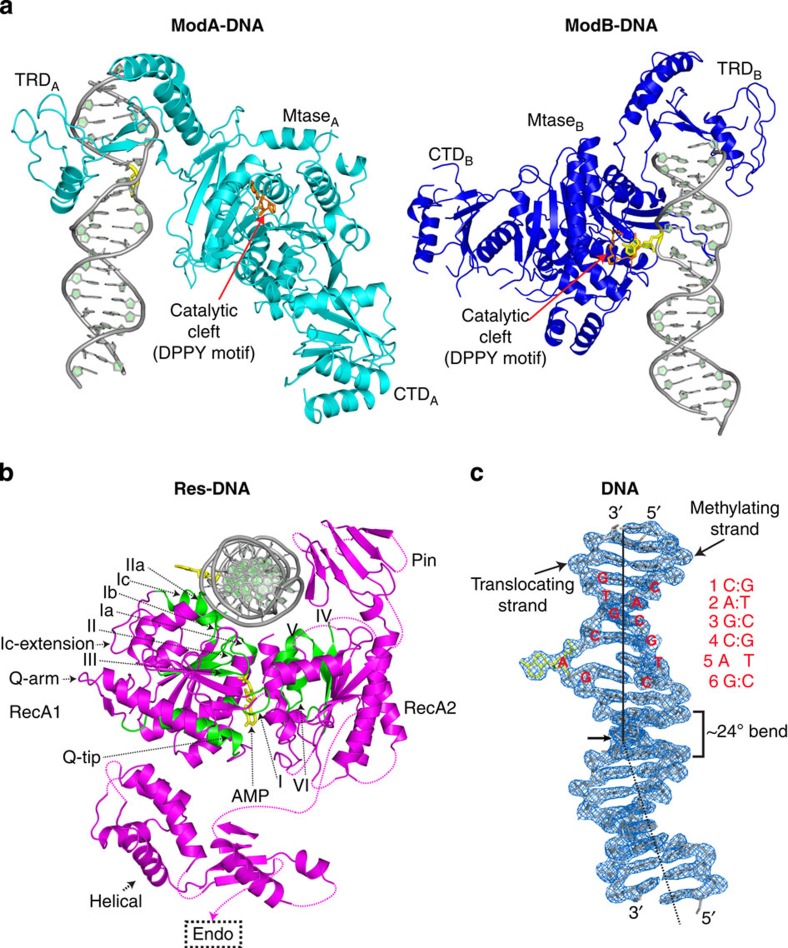
Arrangement of Mod and Res subunits with respect to DNA. (**a**) Division of labour between ModA (cyan) and ModB (blue). ModA inserts its TRD in the DNA major groove, whereas its catalytic cleft (DPPY motif; coloured in orange) in the central MTase domain is located >30 Å away from the target adenine base. ModB accommodates the target adenine (coloured in yellow) in its catalytic cleft, whereas its TRD lies away from the DNA recognition sequence. (**b**) Arrangement of Res (magenta) with respect to DNA (grey). The Res helicase consists of RecA1 and RecA2 motor domains and a helical spacer. Both RecA1 and RecA2 interact with the DNA. RecA2–DNA interactions are augmented by an accessory Pin domain. The classical helicase motifs (I–VI) are highlighted in green. AMP (rendered in yellow) binds in the catalytic cleft between RecA1 and RecA2. (**c**) The DNA is severely distorted. There is an ∼24° bend at the ModA–Res interface (indicated by an arrow) and the target adenine is ejected from the DNA helix. The DNA is shown with electron density from a 2Fo-Fc map (contoured at *σ*=1.7, in blue mash). The DNA recognition sequence (CAGCAG) is labelled and highlighted in red. We refer to the DNA strand containing the target adenine as the methylating strand, and the complementary DNA strand as the translocating strand. The Res subunit makes the majority of DNA contacts with the translocating strand (see for example, Fig. 2b).

Each Mod subunit is composed of four domains, an amino-terminal domain (NTD, aa 14–60), a central methyltransferase domain (MTase, aa 62–262, 390–516), a target recognition domain (TRD, aa 263–384) and a carboxy-terminal domain (CTD, aa 539–644; Fig. 2a,b, Supplementary Fig. 2). The MTase domain contains nine motifs (I–VIII and X) characteristic of amino methyltransferases^34^, and forms the ‘hub' from which the NTD, TRD and CTD fan outwards (Fig. 2a,b). On the basis of the linear order of the motifs, the Mod belongs to the β class of amino methyltransferases^34^, wherein the TRD is inserted between the N-terminal (IV–VIII) and the C-terminal (X and I–III) motifs (Supplementary Fig. 2). The TRD is split into two lobes, separated by two antiparallel β-strands that act as a hinge (Fig. 2a). The proximal lobe is mainly helical (aa 264–300) and, in ModA, interacts primarily with the DNA backbone. The distal lobe (aa 319–376) extends >40 Å from the MTase domain and contains a number of loops, which track the DNA major groove in ModA but mediate protein–protein interactions in ModB (Fig. 2a and Supplementary Fig. 3a). The NTD is composed of helices that intertwine (from ModA and ModB) to form part of the Mod_2_ dimer interface. The dimeric interface is extensive (∼4,000 Å^2^) and lends to the stability of the Mod_2_ dimer and its ability to act as a standalone methyltransferase that asymmetrically methylates the second adenine of its recognition sequence (5′-CAGC**A**G-3′; Supplementary Fig. 3a)^6^. A superposition of ModA and ModB shows an ∼67° movement in the TRD and ∼122° movement in NTD (Supplementary Fig. 4a), which preclude the binding of a second DNA molecule to the Mod_2_ dimer and the binding of a second Res to ModB, respectively. The CTD has a globular α/β substructure that takes on different roles in ModA and ModB. In ModA, the CTD extends towards the Res subunit and makes extensive protein–protein contacts with it, whereas the CTD in ModB is solvent exposed and limited to a few lattice contacts (Fig. 2a, Supplementary Fig. 3b,c and Supplementary Table 2).

The helicase core of the Res subunit is composed of tandem RecA-like domains^14^ (Supplementary Fig. 4b), N-terminal RecA1 (aa 7–269) and C-terminal RecA2 (aa 366–594), followed by a helical spacer (aa 620–810; Fig. 2b). The spacer connects to the endonuclease domain (disordered in the structure). Each RecA-like domain consists of a central β sheet of six to seven parallel β-strands sandwiched by helices. AMP binds to the ‘bottom' side of the cleft at the confluence of two domains, while the DNA duplex is accommodated on the ‘top' side of the cleft (Fig. 2b). The AMP is highly mobile in the structure (B-factor of 139 Å^2^). The helicase motifs typically associated with ATP binding/hydrolysis, interdomain communication and DNA/RNA binding are located on loops that line the cleft (Fig. 2b). Altogether, RecA1 contains the classical motifs Q, I (or Walker A), Ia, Ib, Ic, II (or Walker B), IIa and III, whereas RecA2 contains the motifs IV, IVa, V and VI (Fig. 2b and Supplementary Fig. 5).

The specificity of helicases and translocases for different substrates is dictated to a large extent by accessory domains derived from ‘inserts' in RecA1 or RecA2, or from the N- and C-terminal flanking sequences^14^. In DNA and RNA helicases, for example, an accessory domain can act as a ‘wedge' to disrupt base pairing for the unwinding reaction^14^. In EcoP15I, we identify three new substructures, namely a loop after motif Ic (‘Ic- extension'; aa 198–211), a β-hairpin-like ‘Q-arm', formed by an ∼50aa insertion (aa 28–77) in RecA1, and a more elaborate substructure, ‘Pin' domain, formed by an ∼77aa insertion (aa 288–365) in RecA2 (Supplementary Table 2). The Pin domain adopts a β-sandwich-like tertiary structure with two overlaid β-sheets that extends towards the ModA TRD and interacts with the translocating strand of the DNA duplex (Figs 1 and 2b). The Pin domain is highly mobile (B-factor of 89 Å^2^), but we could assign the main chain and some of the side chains.

### DNA conformation

The DNA is severely distorted from B-form at two sites along its axis (Fig. 2c and Supplementary Fig. 6). First, the site where the target adenine is ejected from the recognition sequence (CAGC**A**G), and second, near the ModA–Res interface (Fig. 2c and Supplementary Fig. 6). For convenience, we refer to the DNA strand containing the target adenine as the ‘methylating' strand, and the opposite strand as the ‘translocating' strand (which makes the majority of contacts with the motor domains—described later; Fig. 2c). The distortions around target adenine and the recognition sequence are mainly induced by the intrusion of ModA TRD in the DNA major groove (Fig. 1). At the ModA–Res junction the DNA is bent ∼24° towards the minor groove, in the direction of the ModA TRD and the Res Pin domain (Figs 1 and 2c). At the site of bending, the torsion angles *ɛ* (C3′–O3′) and ζ (O3′-P) are *gauche*^−^, *trans* rather than more characteristic *trans*, *gauche*^−^ conformation found in B-DNA^35^. Analogous deviations in torsion angles were observed for inner thymines in DNA bound to *Bgl*II, where the DNA experiences an overall bend of ∼23° (ref. 36). Most importantly, the ∼24° bend in the EcoP15I DNA reduces the distance between the ModA TRD and the Res Pin domain to <14 Å and may facilitate an interaction between the two domains when EcoP15I assumes a diffusive or sliding state on DNA (Fig. 1).

### Division of labour: DNA recognition and methylation

The EcoP15I structure is the first of a β-class of an amino DNA methyltransferase bound to DNA and it suggests a fundamentally different mechanism of methylation. There is not only a division of labour between two Mod subunits in terms of DNA recognition (ModA) and methylation (ModB), but also the methylating subunit (ModB) binds to DNA in a radically different manner from other methyltransferases.

**Figure 3:**
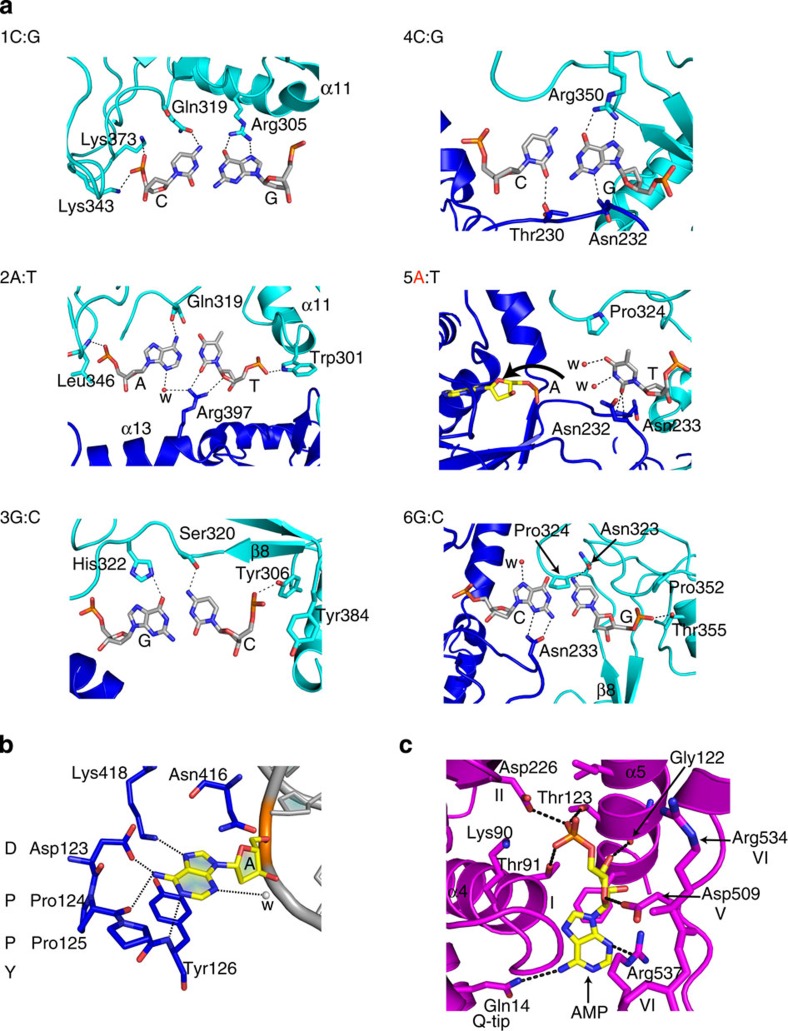
Interactions with DNA and AMP. (**a**) The EcoP15I DNA recognition sequence (CAGCAG) is specified primarily by the ModA TRD (cyan), with a few additional contacts from the ModB-MTase domain (blue). The amino acids that make direct hydrogen bonds (dashed lines) with base pairs 1C:G, 2A:T, 3G:C, 4C:G, 6G:C are labelled. The unpaired thymine of the fifth base pair (5A:T) is stabilized by amino acids and Asn232, Asn233 (ModB-MTase) and Pro324 (ModA TRD) and two water molecules (labelled as w). (**b**) A close-up view of the ModB catalytic cleft containing the conserved DPPY motif. The extrahelical adenine base is accommodated in the cleft and interacts directly with residues Asp123, Pro124, Pro125, Tyr126 that comprise the DPPY motif. Residues Asn416 and Lys418 (from motif X) make additional contacts with the adenine base. (**c**) A close-up view of interactions between AMP and residues from the conserved helicase motifs (Q-tip, I, II, V and VI) that line the interdomain cleft between the RecA1 and RecA2 motor domains.

ModA makes the majority of base-specific contacts, via the bilobed TRD that tracks the DNA major groove and interacts with bases over the entire length of the recognition sequence (Figs 2a,c and 3, Supplementary Fig. 7). In contrast, ModB makes only a few contacts to bases and its role is mainly to methylate the target adenine (CAGC**A**G). The adenine rotates ∼180° out of DNA helix and enters the ModB catalytic cleft (Fig. 2a). In contrast, the ModA catalytic cleft is empty and lies >30 Å away from the DNA (Fig. 2a). Compared with the other amino methyltransferases^27^, ModB pivots around the aspartate/asparagine of the conserved D/NPPY catalytic sequence (motif IV) by >140° so that the ‘PPY' sequence runs along the Watson–Crick edge of the extrahelical adenine base rather than the Hoogsteen edge (Fig. 3b and Supplementary Fig. 8), and the conserved tyrosine (PP**Y**) stacks on opposite face of the base (Supplementary Fig. 8c–e). This unusual mode of DNA docking is a consequence of Mod_2_ dimerization and interactions with the Res subunit, whereby if ModB were to assume the same orientation as say in the monomeric M.TaqI/DNA complex^27^ then ModA would not be in the correct position to recognize the CAGCAG sequence. In addition, the ModA TRD would directly clash with the Res subunit bound to downstream portion of the DNA (Supplementary Fig. 8f).

### The RecA motor domains

**Figure 4:**
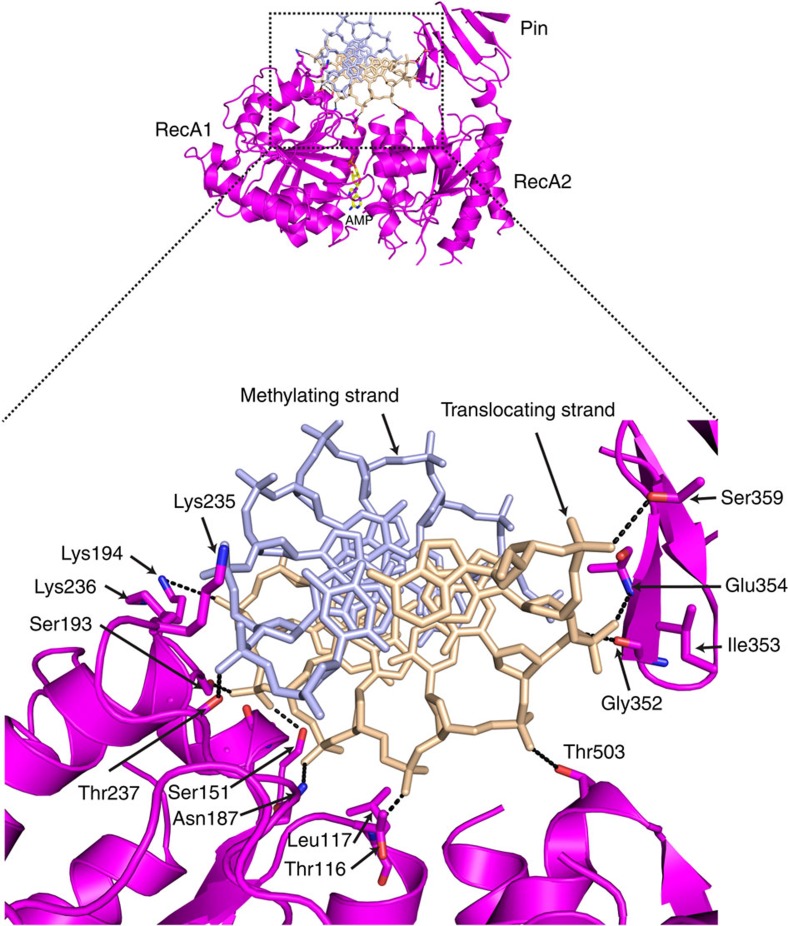
Interactions between the RecA1/RecA2 motor domains and DNA. The DNA binds in the RecA1/RecA2 interdomain cleft—on the opposite side from AMP. Residues on helicase motifs namely Ia (Thr116, Leu117) and Ic (Asn187, Ser193, Lys194) of RecA1 interact with the successive phosphates of the translocating strand (coloured as wheat). Residues on switch loop or motif IIa (Lys235–Thr237) of RecA1 interact with phosphates on the complementary methylating strand (light blue). Most of the contacts to DNA from RecA2 are from the mains chain amides of residues on the accessory Pin domain (Gly352, Ile353, Glu354 and Ser359).

EcoP15I is only the second SF2 translocase (after ssRAd54) to be crystallized with a duplex DNA bound to the RecA motor domains. As with ssRAd54 (ref. 33), the EcoP15I motor domains interact predominantly with one strand of the DNA duplex—the translocating strand (Figs 1 and 4). However, the motor domains in ssRAd54 adopt an unusual arrangement, in which RecA2 is flipped 180^o^ with respect to RecA1, limiting the number of possible interactions with the DNA (Supplementary Fig. 9a)^33^. The EcoP15I motor domains adopt a more canonical configuration, which is intermediate between the fully ‘closed' configuration observed in the SF2 RNA helicase VASA/ssDNA/AMPPNP complex^37^ and the ‘open' configuration observed in zebrafish Rad54 (zRad54)^38^ (Fig. 4) This ‘semi-closed' configuration (16° outward motion of RecA2 when compared with VASA (Supplementary Fig. 9b), for example, appears to represent an intermediate state, following ATP hydrolysis but before AMP dissociation.

The position of the translocating DNA strand in the EcoP15I structure overlays with the ssRNA in VASA and NS3 RNA helicase structures^37^,^39^, reinforcing the notion that contacts primarily to one DNA strand is a conserved feature in different subfamilies of SF2 helicases and translocases (Supplementary Fig. 9a)^14^. Also, as in the VASA complex^37^, the motif Q in RecA1 and motif VI in RecA2 of EcoP15I interact directly with the adenine base of the bound nucleotide (Fig. 3c). Specifically, Gln14 (motif Q) makes direct hydrogen bonds with the N6 amino group of adenine, while Arg537 (the second arginine of the ‘arginine fingers' of motif VI) makes hydrogen bonds with N3 of adenine and O4' of the ribose sugar. In addition, Asp509 (motif V) makes hydrogen bonds with an oxygen of the sugar. One difference is that whereas Arg579 in VASA (the first arginine of the ‘arginine fingers' of motif VI) makes a direct hydrogen bond with the γ-phosphate of AMPPNP, the equivalent residue in EcoP15I (Arg534) points away from the bound AMP due to the absence of γ-phosphate (Fig. 3c).

Both RecA1 and RecA2 interact with the DNA duplex (Figs 1 and 4). The RecA1 residues Thr116 and Leu117 of motif Ia, Ser151 of motif Ib, and Asn187, Met190, Ser193 and Lys194 of motif Ic interact with successive phosphates on the translocating DNA strand, while residues Lys235, Lys236 and Thr237 on the switch II region interact with the opposite methylating DNA strand (Fig. 4). RecA2 interacts with the more downstream portion of the translocating DNA strand via amino acids Thr503 and Arg505 of motif V. In particular, the Pin domain in RecA2 is involved in a number of hydrogen bonds with DNA via the main chain amides of Glu354 and Lys356 and the side chain of Ser359, as well as hydrophobic contacts via Gly352 and Ile353 (Fig. 4). Altogether, the EcoP15I motor domains are specifically adapted to bind double-stranded (ds) DNA. Importantly, there is no equivalent of a ‘wedge' to separate DNA strands of the DNA duplex, but instead the Pin domain in RecA2 augments interactions with the backbone of the translocating DNA strand and which is more apt for diffusion on ds DNA (Fig. 4).

## Discussion

The multisubunit Type I and Type III enzymes are exceptional in their dependency on ATP for restriction activity. By contrast, the Type II enzymes do not require ATP and majority of them harbour functionally independent R and M subunits with the exception of a few enzymes like Type IIG BpuSI or Type IIL MmeI^40^,^41^. Type IV enzymes only cleave modified DNA substrates. The Type III R–M enzymes have defied structural interpretation for >40 years. We report here the first structure of a Type III R–M system, consisting of the entire EcoP15I complex (Mod_2_Res_1_) bound to its DNA substrate. The structure provides unprecedented new insights into the molecular underpinnings of asymmetric DNA methylation and ATP-triggered DNA diffusion.

The early structures of DNA methyltransferases with DNA revealed monomeric enzymes with the ability to both recognize and methylate DNA. This feature extended to both cytosine C5 and adenine N6 methyltransferases^26^,^27^. As such, much of the subsequent data on DNA methyltransferases have been interpreted in a context of a monomer, even in cases where they were observed as dimers^42^,^43^,^44^. All of the current structural information on dimeric DNA methyltransferases is limited to crystal structures in the absence of DNA. The EcoP15I structure provides a mechanistic basis for the action of β amino methyltransferases, which are observed primarily as dimers in solution or in crystals^42^,^43^,^44^. Indeed, it is conceivable that this entire subfamily of DNA methyltransferases works in the same manner as EcoP15I, wherein one subunit recognizes the DNA while the other subunit methylates the target base. The β-amino methyltransferases differ from methyltransferases in other subfamilies in how the TRD is positioned with respect to the MTase domain^34^. In monomeric M.HhaI (α class) and M.TaqI (γ class)^26^,^27^, for example, the TRD is adjacent to the active site cleft and in a direction that permits it to enter the DNA major groove next to the flipped target base. By contrast, in EcoP15I, the TRD lies far off from the active site cleft and in a direction that makes it geometrically impossible for a single Mod subunit to both methylate a target base and recognize the DNA sequence; instead it is reliant on the TRD of the second Mod subunit.

Strikingly, this division of labour may also extend to RNA methylation in mammalian cells^30^. In particular, adenine N6 methylation is the most prevalent modification in the body of nuclear and cytolasmic RNAs in mammals and is implicated in processes ranging from mRNA splicing to translation regulation^30^,^45^,^46^. Intriguingly, adenine N6 methylation of mRNA has also been shown recently to be critical for stem cell commitment and differentiation^31^,^47^,^48^. The modification often occurs in the context of a G(G/A)**A**CU sequence and the enzyme(s) responsible have recently been identified as the METTL3/METTL14 heterodimer^49^,^50^,^51^. Intriguingly, both METTL3 and METTL14 contain motifs (X, I–VIII) characteristic of amino methyltransferase^52^, including the equivalent of the ‘DPPY' sequence in motif IV (DPPW in METTL3 and EPPL in METTL14). The linear order of these motifs suggests that both METTL3 and METTL14 belong to the β-amino class of methyltransferases and—based on the EcoP15I structure—it is conceivable that one methyltransferase (METTL3 or METTL14) plays a more dominant role in recognition of the G(G/A)ACU sequence (and perhaps also the surrounding RNA secondary structure), while the other plays a more central role in adenine methylation.

The EcoP15I methylation mechanism may also extend to other subfamilies of DNA methyltransferases, such as the plant *de novo* DNA methyltransferase DRM2, which functions as a homodimer^28^. On the basis of the EcoP15I structure, one can envisage a mechanism where one DRM2 monomer recognizes the DNA sequence context (CG/CHG/CHH, where H=A, T, or C) while the other methylates the target cytosine. The dimeric RNA MTases from SPOUT family display another form of division of labour in which the RNA binds in a cleft between the two monomers, whereas the target RNA base for methylation resides in the catalytic pocket of one monomer^53^,^54^,^55^. Altogether, a division of labour between two or more methyltransferase subunits appears to be a more general mechanism in DNA and RNA methylation. The EcoP15I structure provides a framework for beginning to understand the interplay between different methyltransferase subunits.

As recently as 1993, helicases were considered as DNA- or RNA-unwinding machines that couple ATP hydrolysis for the unwinding reaction^56^. The discovery that many helicases are actually translocases (especially those in the SF2 superfamily) has changed this view^14^; however, even this view has been found wanting with the discovery that several helicases or translocases behave as molecular switches^21^. These molecular switches have also been referred to as pseudo-helicases^19^, wherein ATP hydrolysis is coupled to a conformational change in the enzyme for thermally driven diffusion on the DNA or RNA. The EcoP15I structure uncovers a helicase motor that is generally similar to that observed in classical helicases and translocases, composed of tandem RecA-like domains with Walker A and B motifs and an arginine finger, among other classical motifs^14^.

**Figure 5:**
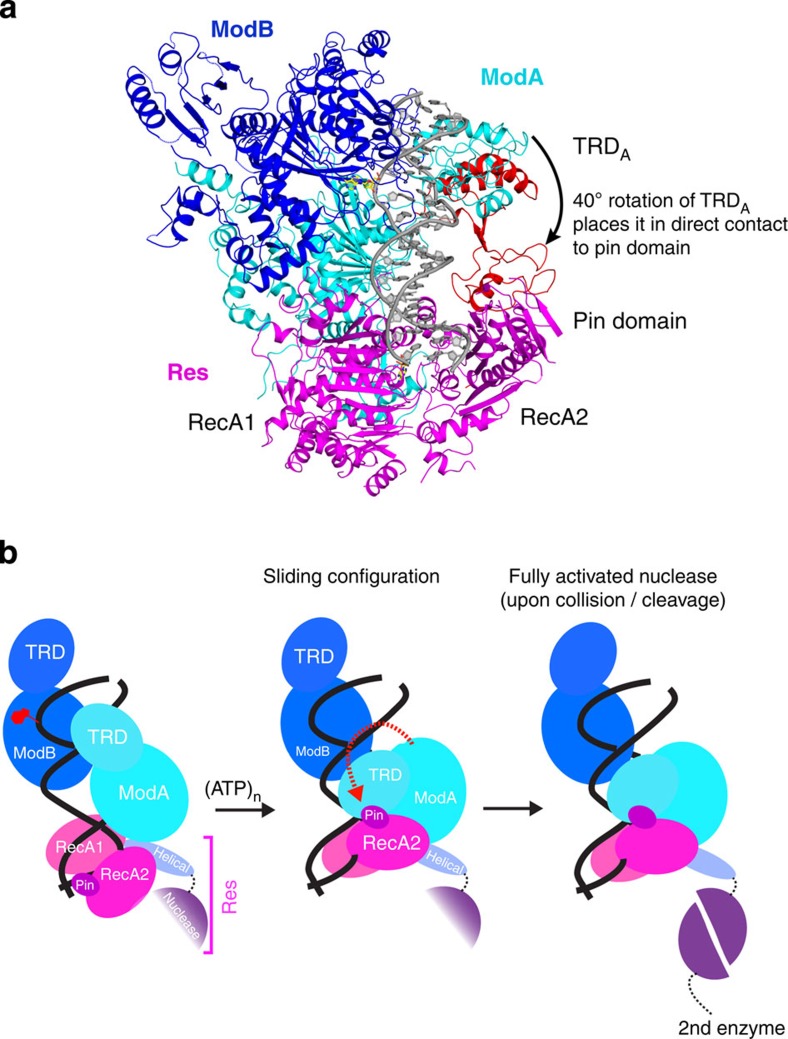
A model for DNA sliding. (**a**) A simple 40° rotation of TRD_A_ around the hinge region places it in direct contact with Pin domain—sequestered away from the DNA. (**b**) Hypothetical structural changes in the Mod and Res subunit that initiate DNA sliding, followed by DNA cleavage.

What is the nature of the ATP-triggered conformational change for diffusion on DNA? The structure here provides some interesting clues. In particular, proximity of the ModA TRD to the Pin domain in RecA2 suggests a model in which the TRD may switch its location from the DNA major groove to the Pin domain and hence, adopt a ‘nonspecific' conformation more amenable to DNA sliding^19^ (Fig. 5). The TRD is joined to the MTase domain by a flexible linker, and a simple rotation of ∼40° about this linker puts the ModA TRD in direct contact with the Pin domain—sequestered away from the DNA (Fig. 5a). Moreover, the DNA duplex is bent by ∼24° at this precise ModA–Res nexus, which reduces the distance between the TRD and the Pin domain to <14 Å. The Pin domain is highly mobile and may only become fully ordered when it recruits the ModA TRD. The asymmetric nature of ModA and ModB DNA binding seems to ensure that only one TRD (and not both) needs to be drawn away from the DNA. Overall, the structural model is in accord with single-molecule studies, which suggest that the entire Mod_2_Res_1_ complex diffuses on the DNA (and not just the Res subunit) until it collides with another complex to become cleavage competent (Fig. 5b, Supplementary Fig. 1)^19^.

In conclusion, we present here the first structure of a Type III R–M system, consisting of the entire EcoP15I complex (Mod_2_Res_1_) bound to its DNA substrate. Asymmetric methylation and ATP-triggered DNA diffusion are emerging themes in the study of methyltransferases and helicases but the mechanisms remain unclear. Plant DRM2 homodimer^28^ and the mammalian METTL3/METTL14 heterodimer^30^,^45^,^46^,^49^,^50^,^51^, for example, may operate in a similar manner to EcoP15I, where one monomer recognizes the DNA or RNA sequence context, while the other methylates the target base. Neither DRM2 nor METTL3/METTL14 possesses helicase activity. Furthermore, an EcoP15I type DNA sliding-based mechanism has also been proposed for the mismatch repair protein MutS (and its eukaryotic homologue), but where nucleotide exchange (rather than hydrolysis) triggers sliding on DNA after mismatch recognition^22^,^23^,^24^. Similarly, the loading of processivity clamps at replication forks by clamp loaders occurs via a two-step conformational change mediated by ATP binding^57^. Altogether, the EcoP15I structure proffers unprecedented new insights into the molecular underpinnings of asymmetric DNA/RNA methylation and ATP-triggered thermal diffusion in broad array of DNA and RNA metabolism.

## Methods

### Expression and purification

The genes encoding Res and Mod subunits of EcoP15I were subcloned from a plasmid kindly provided by Dr D.N. Rao (Indian Institute of Science) into an expression vector pRRS^58^. *E. coli* expression host NEB Express (NEB) was transformed and was grown in LB medium containing 100 μg ml^−1^ of ampicillin. Protein expression was carried out for 18 h at 30 °C. The harvested cells from 6 l of culture were lysed and the derived cell pellet was suspended in a potassium phosphate buffer (20 mM potassium phosphate, pH 7.0, 50 mM NaCl, 5% Glycerol) and sonicated on ice. The lysate was centrifuged at a maximum r.c.f. of 31,000*g* for 30 min at 4 °C and the supernatant was loaded onto a heparin column. The bound proteins were eluted using a NaCl gradient. Fractions containing EcoP15I activity were pooled and loaded onto a ceramic hydroxylapatite column (Bio-Rad; 7 ml), followed by elution with a potassium phosphate gradient. Fractions containing EcoP15I activity were pooled and loaded onto a cation exchange column and eluted using a NaCl gradient. Peak fractions were pooled, and concentrated using a Vivaspin 15 concentrator (10 KDa MWCO; Sartorius Stedim Biotech) to a final concentration of >10 mg ml^−1^.

### Crystallization

We co-crystallized EcoP15I complex in presence of a 20-mer DNA duplex and AMP. The crystals were obtained in a hanging drop set up by mixing 1 μl of EcoP15I/DNA/AMP complex with 1 μl of precipitant solution containing 10% PEG 5000 monomethyl ether, 0.1 M HEPES pH 7.5, 0.2M potassium acetate and 15 mM MnCl_2_ at 20 °C, and were cryoprotected by serial transfer into mother liquor containing 30% PEG 5000 MME and 10% PEG400 before plunging them into liquid N_2_. Neither *S*-adenosyl methionine (AdoMet) nor its analogue AdoHcy was included during purification or crystallization. The crystals belong to the space group P4_1_2_1_2 with unit cell dimensions of *a*=*b*=101 Å, *c*=533 Å and *α*=*β*=*γ*=90°. X-ray diffraction data were measured at beamlines NECAT-24IDC at Advanced Photon Source (APS), and X4A, X25 and X29 at NSLS of Brookhaven National Laboratory (BNL; Table 1).

### Structure determination

To calculate the experimental phases for structure determination, we used X-ray data from native crystals and seven heavy atom derivatives (Se, Br, I, Ta, Sm, Co, Ho). The phases were calculated by the MIRAS method, using the programme SHARP^59^. The bromine and iodine derivatives were prepared by substituting 7 and 8 thymines (outside of the recognition sequence in the 20-mer DNA duplex) to 5-bromouracils and 5-iodouracils, respectively. The Se-Met-labelled protein was expressed using standard method^60^ and purified with similar protocol as the WT enzyme. The tantalum (Ta), cobalt (Co) and holmium (Ho) derivatives were prepared by soaking native crystals into the mother liquor containing 1 mM hexatantalum tetradecabromide (for 22 h), 15 mM cobalt chloride (for 16 h), 2 mM holmium sulfate (16 h), respectively. The samarium (Sm) derivatives were prepared by co-crystallizing the EcoP15I complex in presence of 0.5 mM samarium acetate. The single wavelength anomalous X-ray data were measured at wavelengths close to the absorption K edge for Se (0.9792 Å), Co (1.60 Å) and Br (0.9197 Å) derivatives, and the L-III edge for the Ta (1.255 Å) and Sm (1.849 Å) derivative. X-ray data for the iodine derivative were measured at wavelength of 1.608 Å. All the data were processed using processed using the programme autoProc^61^. The MIRAS phases and solvent-flattened maps were calculated using SHARP^59^, and the model was built manually using programme COOT^62^ and refined using programme BUSTER^63^. Among all heavy atom derivatives, the Se-Met data set gave the best phases (anomalous phasing power ∼1.0 at 5.85 Å). At a later stage, another X-ray data set on the 5-iodouracil containing crystals was measured at a longer wavelength (2.07 Å) on five of such crystals and processed, merged, and scaled using the XDS programme package^64^ (Table 1). These data were used for molecular replacement-single wavelength anomalous diffraction phasing to generate log-likelihood-gradient maps in programme Phaser^65^ in CCP4 that were used at a late stage of model building. These log-likelihood-gradient maps also confirmed the location of heavy atoms S, I and P. The model was improved through iterative cycles of density modification in presence of model, followed by manual rebuilding and refinement (Supplementary Fig. 10). The final model was refined to 2.6 Å resolution with *R*_free_ and *R*_work_ values of∼26.4% and 21.9%, respectively (Table 1).

## Additional information

**Accession codes**: Atomic coordinates and structure factors have been deposited in the Protein Data Bank under accession codes 4ZCF.

**How to cite this article:** Gupta, Y. K. *et al*. Structural basis of asymmetric DNA methylation and ATP-triggered long-range diffusion by EcoP15I. *Nat. Commun*. 6:7363 doi: 10.1038/ncomms8363 (2015).

## Supplementary Material

Supplementary Information Supplementary Figures 1-10, Supplementary Tables 1-2 and Supplementary References

## Figures and Tables

**Table 1:** Data collection, phasing and refinement statistics.

		EcoP15I/DNA/AMP	Se	I	Br	Ta	Co	Sm	I*
*Data collection*									
Space group		P4_**1**_2_**1**_2	P4_**1**_2_**1**_2	P4_**1**_2_**1**_2	P4_**1**_2_**1**_2	P4_**1**_2_**1**_2	P4_**1**_2_**1**_2	P4_**1**_2_**1**_2	P4_**1**_2_**1**_2
Cell dimensions (Å)									
*a*=*b*, *c*		101, 535	101.7, 534.8	101.6, 535.6	100.8, 532.5	101, 536.3	100.4, 534.1	101, 531.9	101.6, 533.4
Wavelength (Å)		0.9795	0.9792	1.608	0.9197	1.255	1.6057	1.7712	2.07
Resolution (Å)		2.6	3.4	3.0	2.9	3.9	3.25	2.74	3.0
*R*_sym_ (%)†‡		11.5 (72.1)	10.6 (68)	10.3 (79.5)	13.1 (95.7)	8.6 (59)	6.2 (49)	12 (51)	
*R*_merge_ (%)§		—	19.3	21.8	13.6	9.7	25.2	16.5	25.3
Number of reflections		85,834 (8063)	39,994	56,204	63,900	25,925	44,187	74,060	57,716
Completeness (%)		97.3 (94.5)	98.5 (97)	100 (100)	100 (99.9)	99.5 (99.9)	98 (91)	100 (99.9)	100 (100)
Redundancy		15.1 (15.0)	4.4 (2.8)||	5.1 (4.2)||	5.6 (5.2)||	2.8 (2.2)||	6.9 (4.3)||	7.4 (5.2)||	61.3 (23.2)||
Mean ((*I*)/(s.d.(*I*))		15.5 (3.4)	12.6 (2.2)	17.3 (2.4)	15.7 (2.1)	10.7 (2)	18 (2.2)	19.1 (2.9)	29.4 (2.7)
									
*Phasing method*	MIRAS								
Number of sites			34	8	7	2	1	1	
Acentric/centric mean figure of merit	(0.19/0.2)								
									
*Refinement*									
Resolution (Å)		40–2.6							
*R*_factor_ (%)¶/*R*_free_ (%)#		21.9/26.4							
Non-hydrogen atoms									
Protein/DNA/AMP/water		14,003/818/124/103							
Average B-factors (Å^2^)									
Protein and DNA/AMP/water		60.0/139.0/42.3							
r.m.s. deviations									
Bond lengths (Å)		0.010							
Bond angles (^o^)		1.25							
Ramachandran plot									
Most favoured (%)		94							
Additional allowed (%)		5.4							
Outliers (%)		0.5							

MIRAS, multiple isomorphous replacement with anomalous scattering; r.m.s., root mean squared.

^*^Iodine data measured at 2.07 Å wavelength on five crystals and merged together.

^†^Values for outermost shells are given in parentheses.

^‡^*R*_sym_=Σ|*I*−<*I*>|/Σ*I*, where *I* is the integrated intensity of a given intensity.

^§^*R*_merge_ represents weighted *R* factor of individual heavy atom data sets against the native data in MIRAS calculation in programme SHARP.

^||^Anomalous multiplicity.

^¶^*R*_factor_=Σ||F_observed_|−|F_calculated_||/Σ|F_observed_|.

^#^*R*_free_ was calculated using 5% of random data omitted from the refinement of EcoP15I/DNA/AMP complex.
