# Supplementary material for: Structural basis of asymmetric DNA methylation and ATP-triggered long-range diffusion by EcoP15I
Source: Nat Commun. 2015 Jun 12;6:7363. doi: 10.1038/ncomms8363 (PMC4490356; doi:10.1038/ncomms8363)
Supplement: Supplementary Information — Supplementary Figures 1-10, Supplementary Tables 1-2 and Supplementary References [file ncomms8363-s1.pdf]

Supplementary figure 1

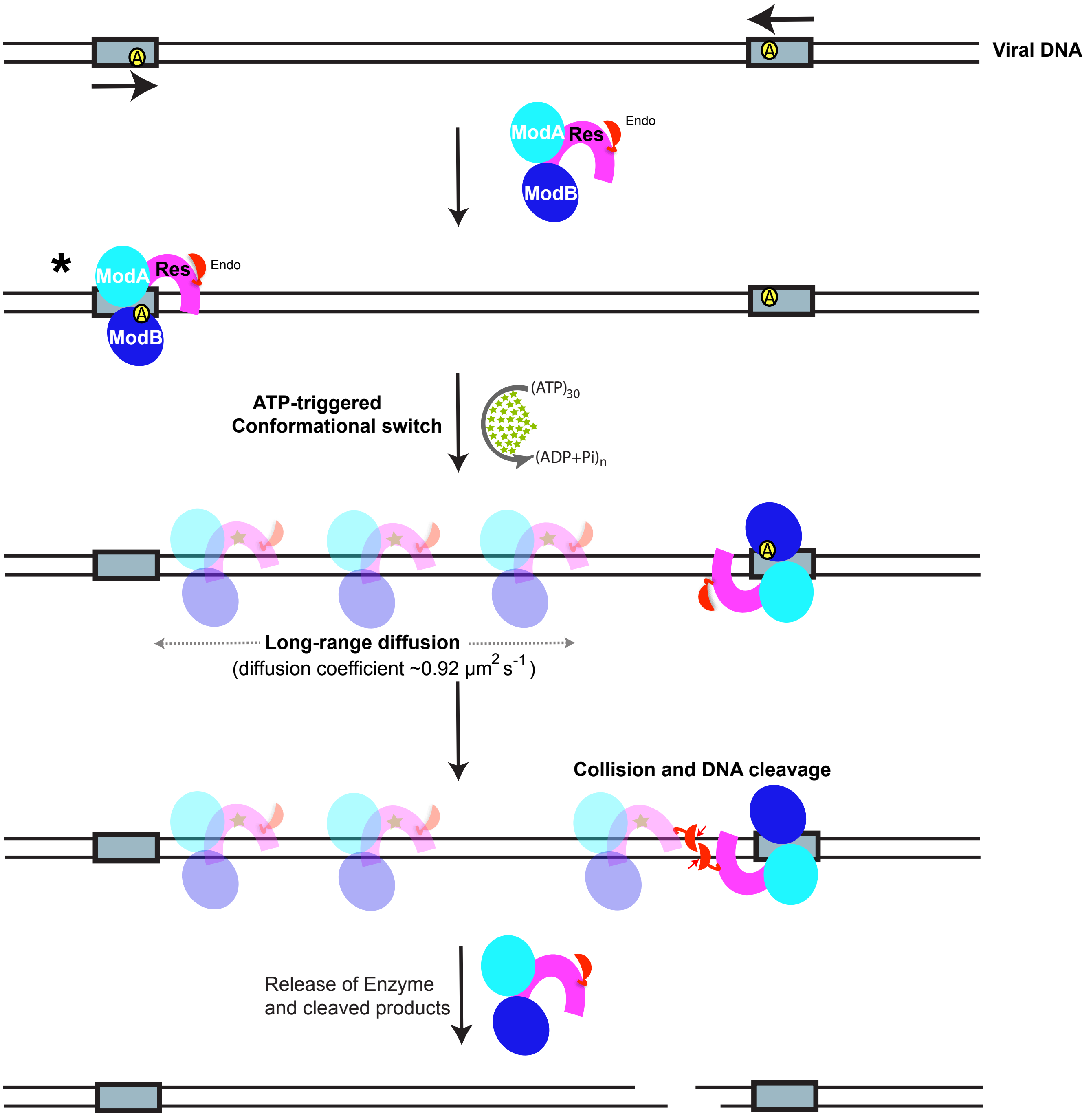

**Supplementary Figure 1. Scheme of EcoP15I action on viral DNA**

EcoP15I binds to recognition sequence CAGCAG, shown here in head-to-head grey boxes. One of the EcoP15I Mod subunits (ModB) accommodates the target adenine base (shown in yellow circle) for methylation, while the other subunit (ModA) recognizes DNA. ATP hydrolysis by the Res subunit switches EcoP15I into a diffusive (sliding) state. The sliding enzyme collides with a second site bound enzyme, converting into a fully active conformation for a double stranded cleavage in the foreign viral DNA, 25/27 bases away from one of the two sites. The state marked by an asterisk represents the structure reported here.

Supplementary figure 2

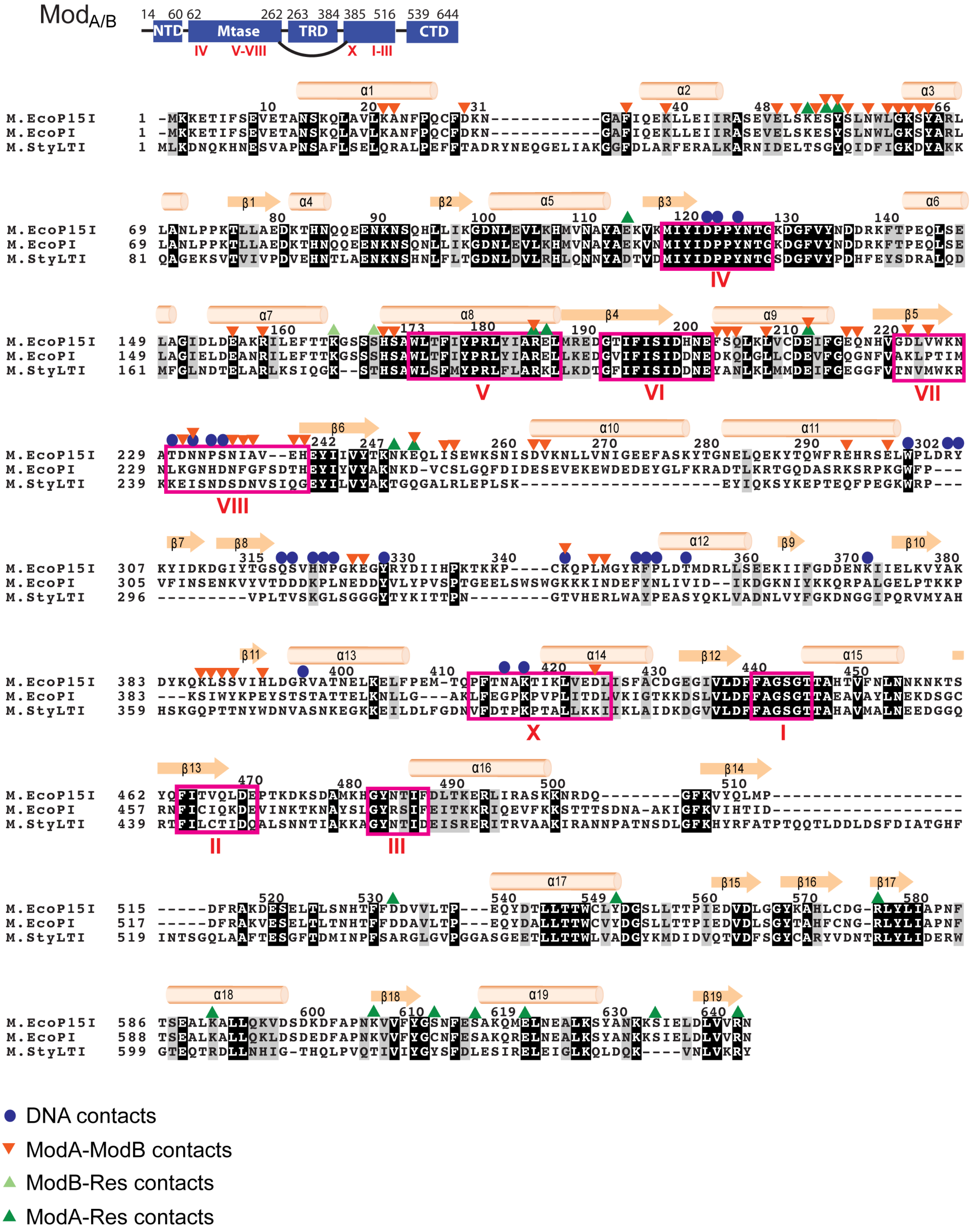

**Supplementary Figure 2. Structural elements of EcoP15I's Mod subunits**

The Mod subunit is a representative of β-class of DNA amino-Mtases. The TRD is inserted in the Mtase core with the classical methyltransferase specific motifs I-III and X reside at the c-terminus whereas motifs IV-VIII locate at the N-terminus portion of Mtase core. The secondary structural elements are shown above the aligned protein sequences from three members of Type III R-M family. Boxes in purple highlight the classical methyltransferase motifs. The approximate boundaries of these motifs are based on the sequence comparisons with other members of β-class described earlier [1]. The different symbols on top of residues represent their respective involvement in inter-subunit interactions (green and red triangles), and DNA binding (blue circles).

Supplementary figure 3

A

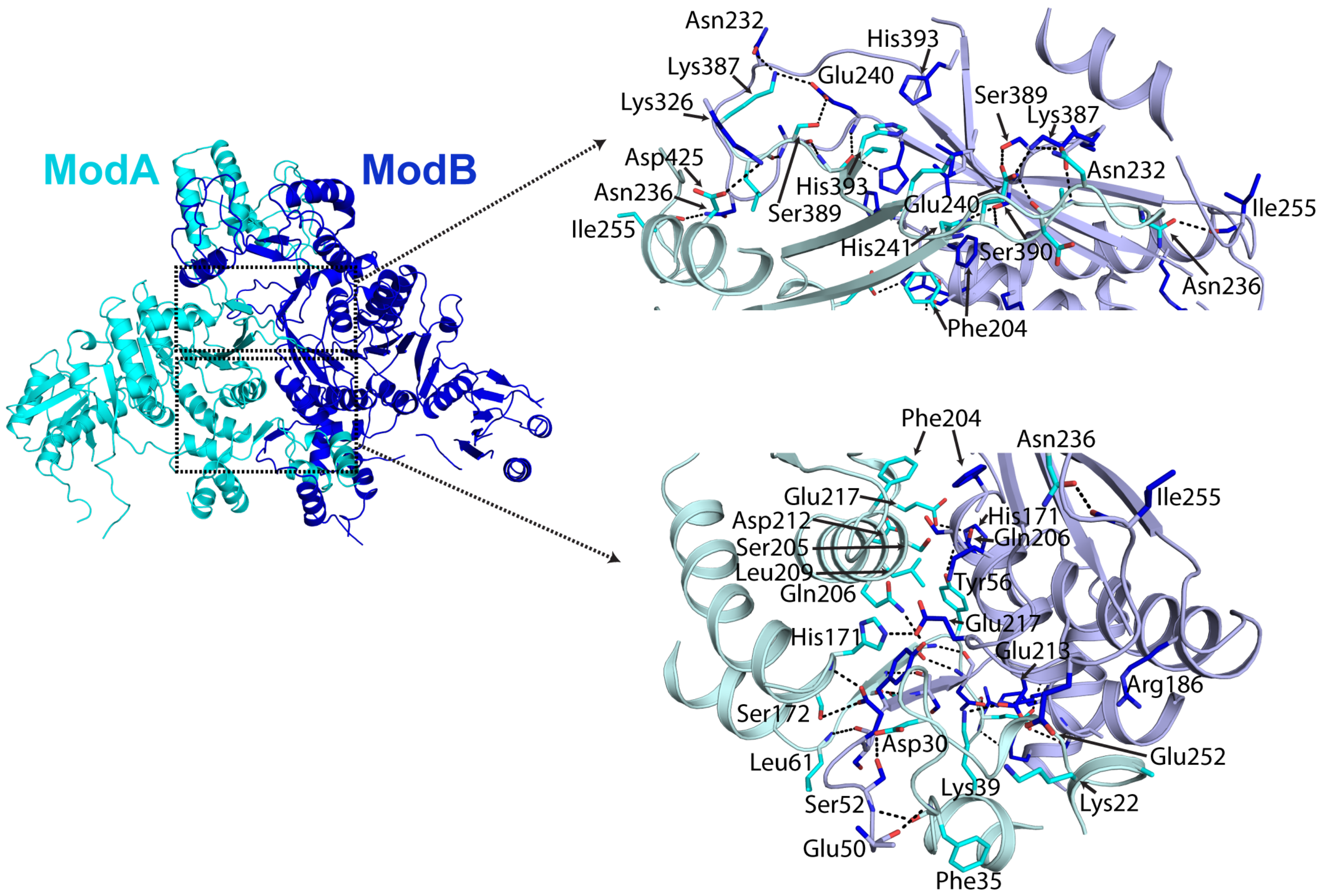


B

Cross talk ModA CTD and RecA1/RecA2

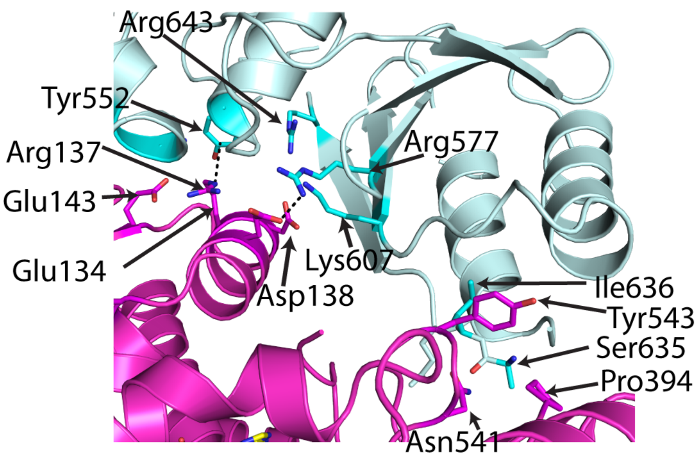


C

Cross talk ModA CTD and helical spacer (Res)

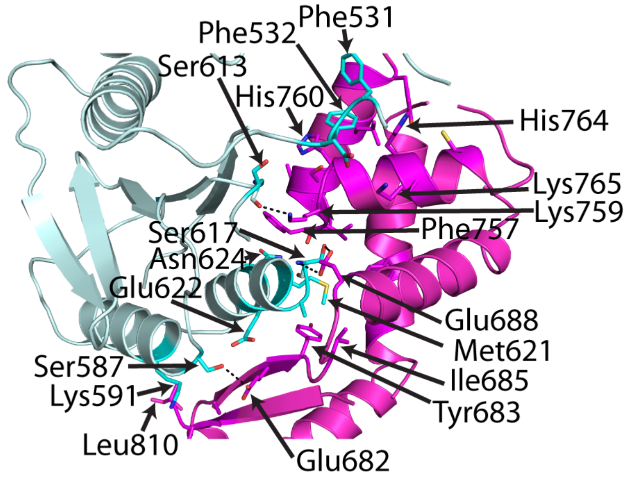

**Supplementary Figure 3. Crosstalk between ModA-ModB and ModA-CTD-Res**

A) ModA (cyan) and ModB (blue) dimerizes through their Mtase and NTD domains. Two close up views of the dimeric interface and the residues from ModA and ModB that engage in both electrostatic and hydrophobic interactions at this dimeric interface are shown in two panels on right. Hydrogen bonds are depicted as black dashes.

Lower panel shows the cross talks between ModA CTD and two RecA domains of Res (B), and helical spacer of Res (C).

a

**Superposition of ModB MTase (aa 62-227) on to ModA**

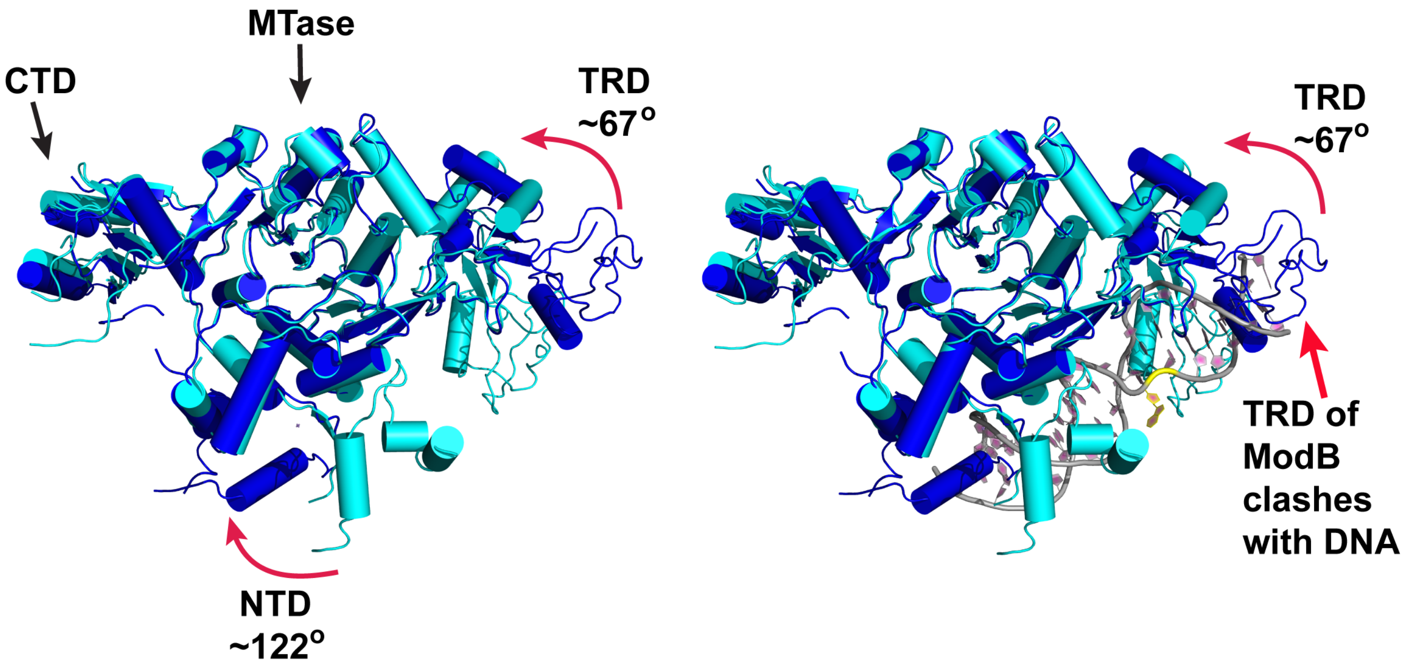


b

**Superposition of RecA1 and RecA2**

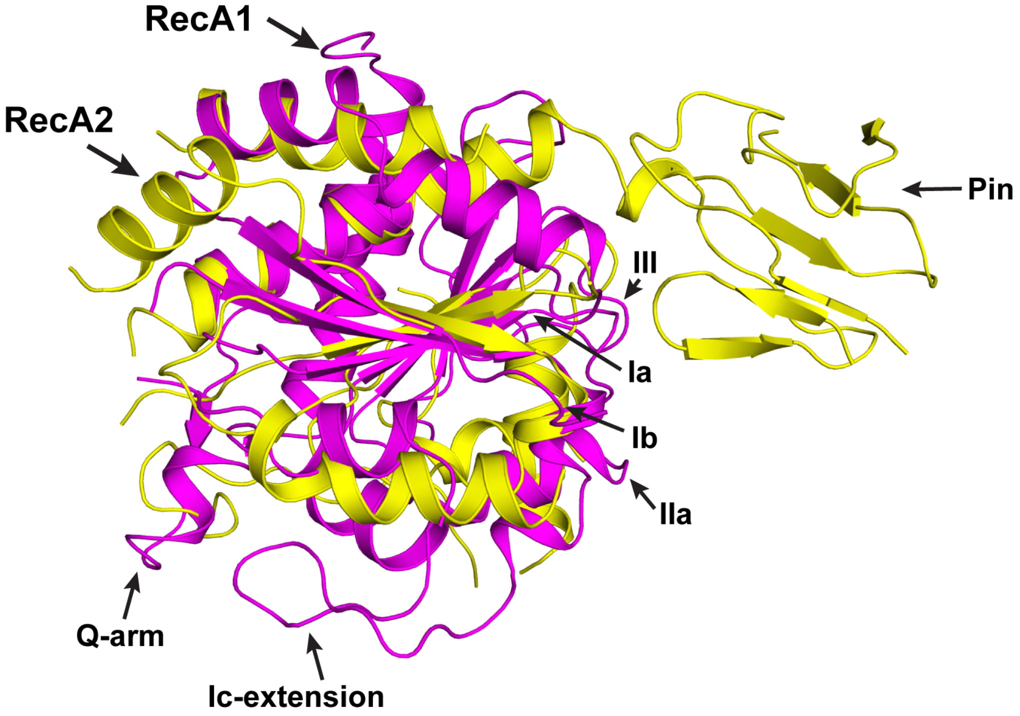

**Supplementary Figure 4. Structural comparison of Mod subunits and RecA motifs of Res**

A) Superposition of Mod B (blue) on Mod A (cyan). This MTase (aa 62-227) based superposition shows that the MTase and CTD domains overlay well whereas the TRD and NTD domains of Mod B rotate from their respective orientations in ModA. The Mod B TRD would clash with the DNA bound to ModA subunit whereas its NTD rotates towards and approaches to its own CTD and therefore likely precludes the binding of a second Res subunit to Mod B. B) Superposition of RecA1 (magenta) and RecA2 (yellow) based on common RecA motifs. The rmsd between the two RecA cores (excluding the Pin domain) is ~3.2Å.

Supplementary figure 5

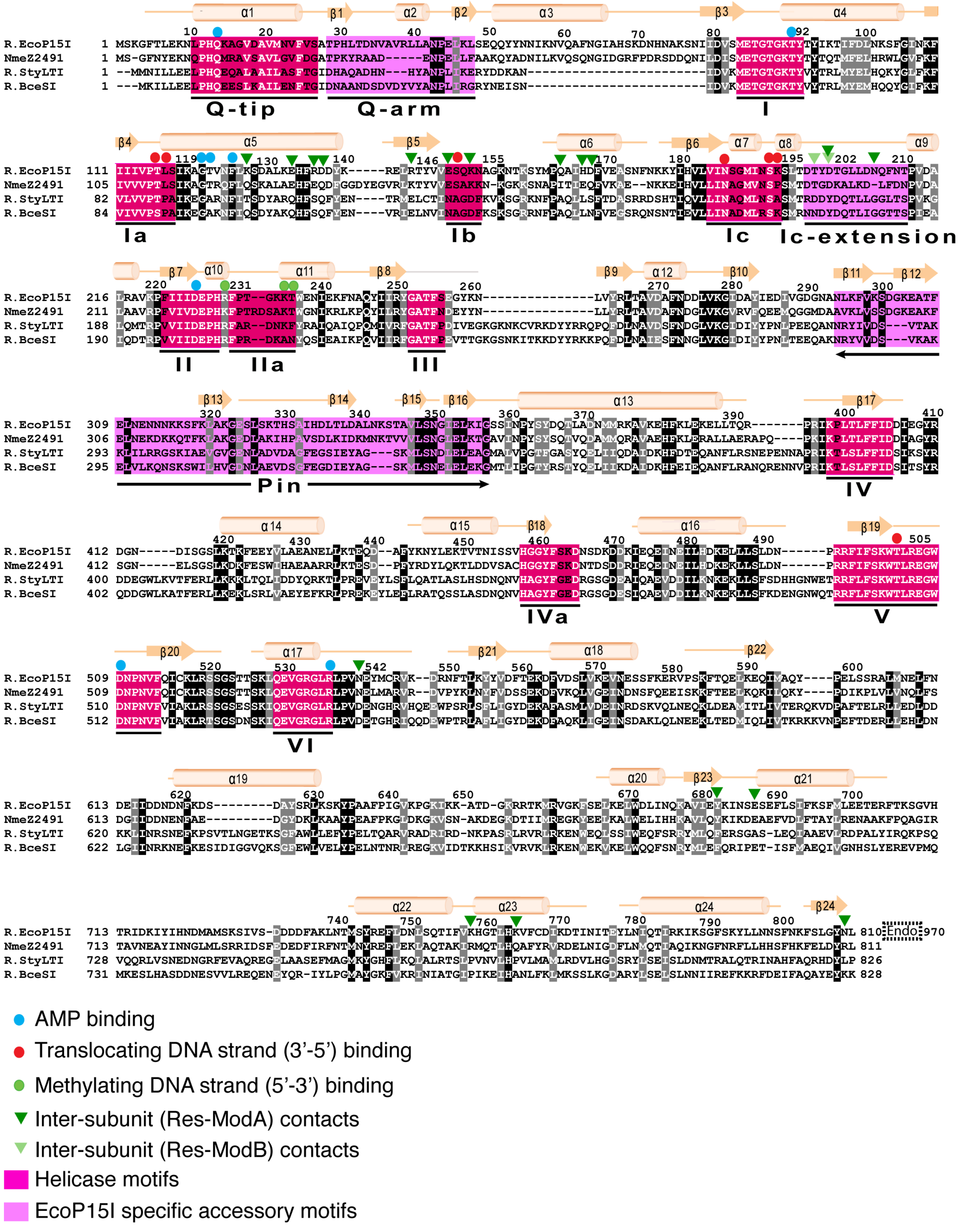

**Supplementary Figure 5. Structural elements of EcoP15I's Res or motor subunit**

The secondary structure of Res subunit is shown above the aligned protein sequences from four members of Type III R-M family. The classical helicase motifs from the N-terminus RecA1 (motifs Q, I, Ia, Ib, Ic, II, and III) and the c-terminus RecA2 (motifs IV-VI) are highlighted in red [2]. The accessory motifs specific to EcoP15I helicase domain (motifs Q-arm, Ic-extension and Pin domain) are highlighted in magenta. The different symbols placed on top of aligned residues represent their respective involvement in inter-subunit interactions (green triangles), DNA binding (red and green circles), and AMP binding (blue circles).

Supplementary figure 6

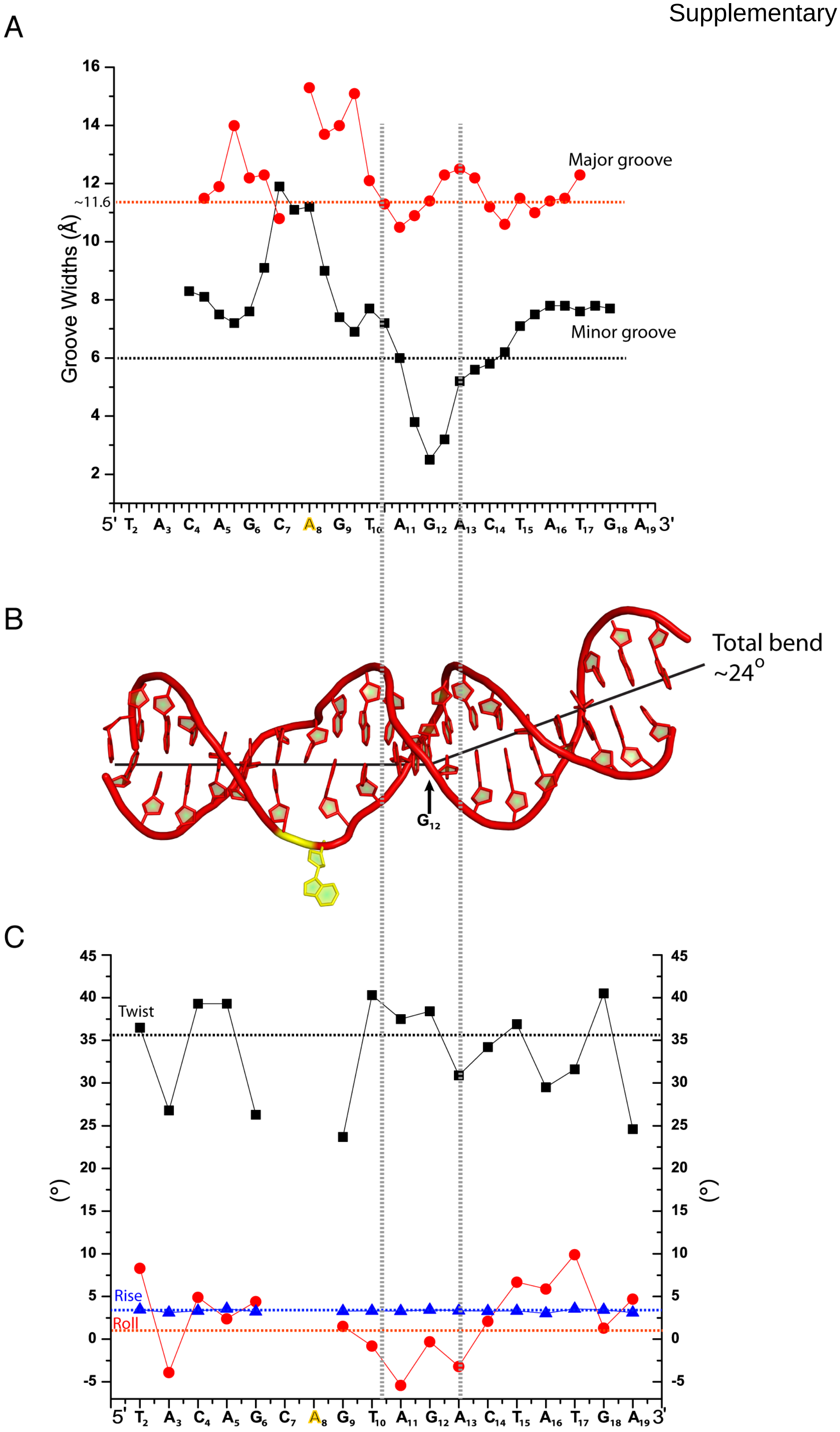

**Supplementary Figure 6. Protein induced distortions in DNA**

A) The upper most panel shows the changes (widening and narrowing) in both major and minor grooves along the DNA axis. The majority of changes in DNA conformation occur at two places; first, around the extrahelical target adenine base at the 5'end wherein ModA TRD enters and widens the major groove, secondly, at the point of $Mod_2$-Res junction in the middle of the sequence where a 24° bend (B) towards the minor groove (suggested from negative roll values, C) occurs and further narrows the minor groove around this area shown by vertical gray lines across all panels (A, B, C). The sum of van der Waals radii (5.8 Å) of the phosphate groups is not added in groove widths. The default values for roll, rise, twist angles, and groove widths in standard B-DNA are shown by horizontal colored lines in the respective graphs. Only the bases from the methylating strand are shown in plots A, and C with flipped out adenine is colored in yellow.

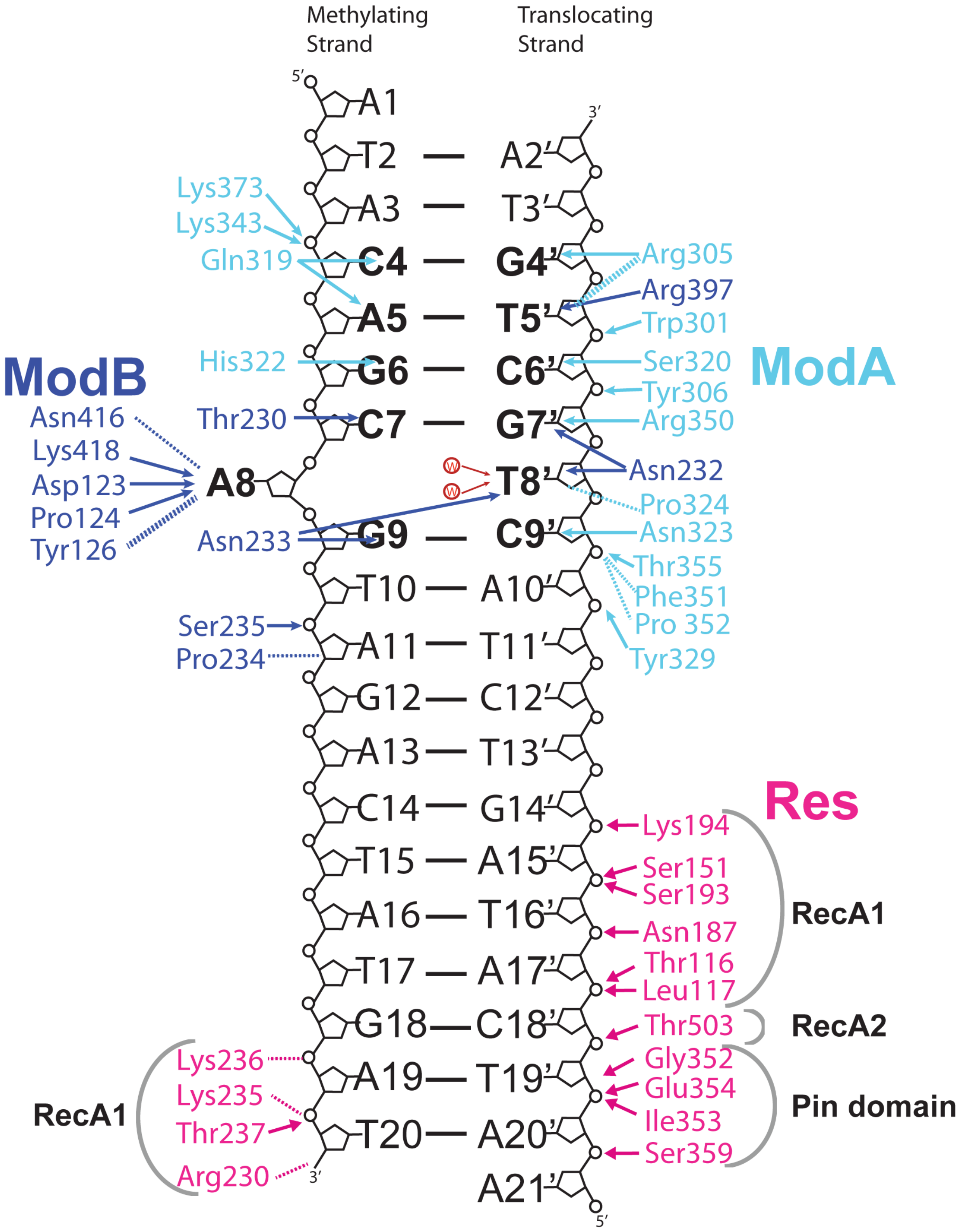
Methylating Strand
Translocating Strand
5′
A1
3′
T2 — A2′
A3 — T3′
C4 — G4′
A5 — T5′
G6 — C6′
C7 — G7′
A8
T8′
G9 — C9′
T10 — A10′
A11 — T11′
G12 — C12′
A13 — T13′
C14 — G14′
T15 — A15′
A16 — T16′
T17 — A17′
G18 — C18′
A19 — T19′
T20 — A20′
3′
A21′
5′
ModB
Asn416
Lys418
Asp123
Pro124
Tyr126
Lys373
Lys343
Gln319
His322
Thr230
Asn233
Ser235
Pro234
ModA
Arg305
Arg397
Trp301
Ser320
Tyr306
Arg350
Asn232
Pro324
Asn323
Thr355
Phe351
Pro 352
Tyr329
W
W
Res
Lys194
Ser151
Ser193
Asn187
Thr116
Leu117
RecA1
Thr503
RecA2
Gly352
Glu354
Ile353
Ser359
Pin domain
Lys236
Lys235
Thr237
Arg230
RecA1

**Supplementary Figure 7. Schematic of protein-DNA interactions**

Residues involved in direct contacts to DNA are labeled with following color scheme, ModA, cyan; ModB, blue; Res, magenta. The six base pair recognition site is shown in bold text. The pointed arrows represent direct hydrogen bonds to either DNA base or phosphates and dashed lines indicate stacking/hydrophobic interactions. Two water molecules stabilize the unpaired T8' base opposite the extrahelical target adenine (A8) base are shown as w in red circles. The DNA strand that harbors the target adenine is labeled as methylating strand whereas the opposite strand that contacts Res subunit at its downstream portion is labeled as translocating strand.

Supplementary figure 8

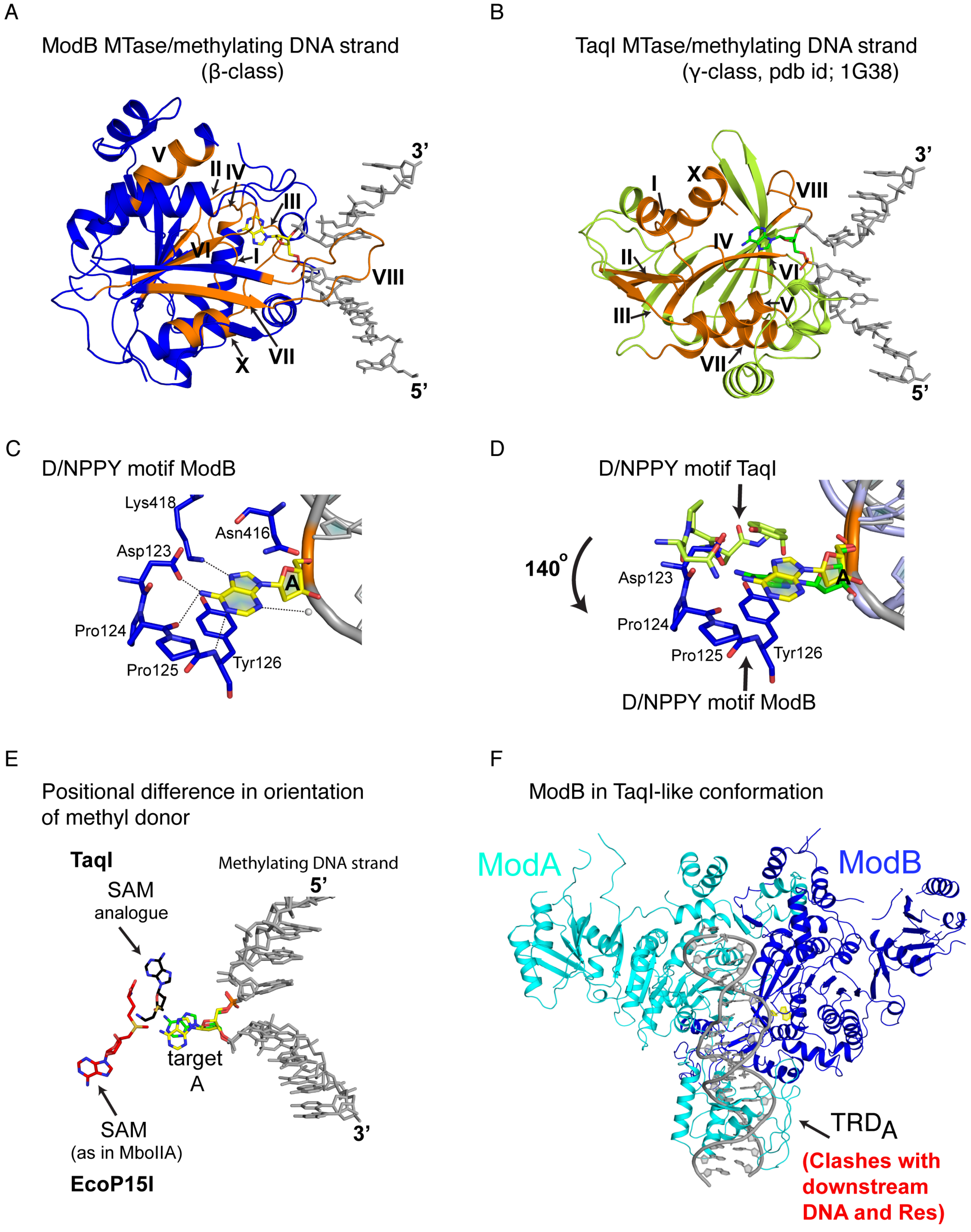

**Supplementary Figure 8. A novel orientation of the methyltransferase core of EcoP15I around the target adenine base**

Superposition based on the extrahelical target adenine in EcoP15I (A) and TaqI (B) shows different orientations of Mtase core in two structures with all the methyltransferase (Mtase) specific motifs (I-X, in orange) orients differently with respect to the flipped out adenine. The methylating strands in both structures overlay very well (E), it is the Mtase core of EcoP15I that rotates ~140° around the target adenine. This 140° rotation of ModB's Mtase core places the "D/NPPY" catalytic motif (blue sticks in EcoP15I and green sticks in TaqI) at the Watson crick edge of adenine base in EcoP15I and at the Hoogsteen edge in TaqI (C, D). The methyl donor SAM (red stick) modeled in ModB using MboIIA Mtase and a SAM analogue (black stick) in TaqI assumes different relative orientation in EcoP15I and TaqI (E). The requirement of Mod dimerization and its binding to Res has necessitated the ModB to assume this significantly different orientation. ModA TRD would otherwise clash with the downstream portion of DNA and Res if ModB to assume TaqI like orientation around the target adenine base (F).

Supplementary figure 9

A semi-closed orientation of EcoP15I's RecA domains around the translocating DNA strand (5'-3')

A Superposition of walker A motifs of EcoP15I vs ssoRad54

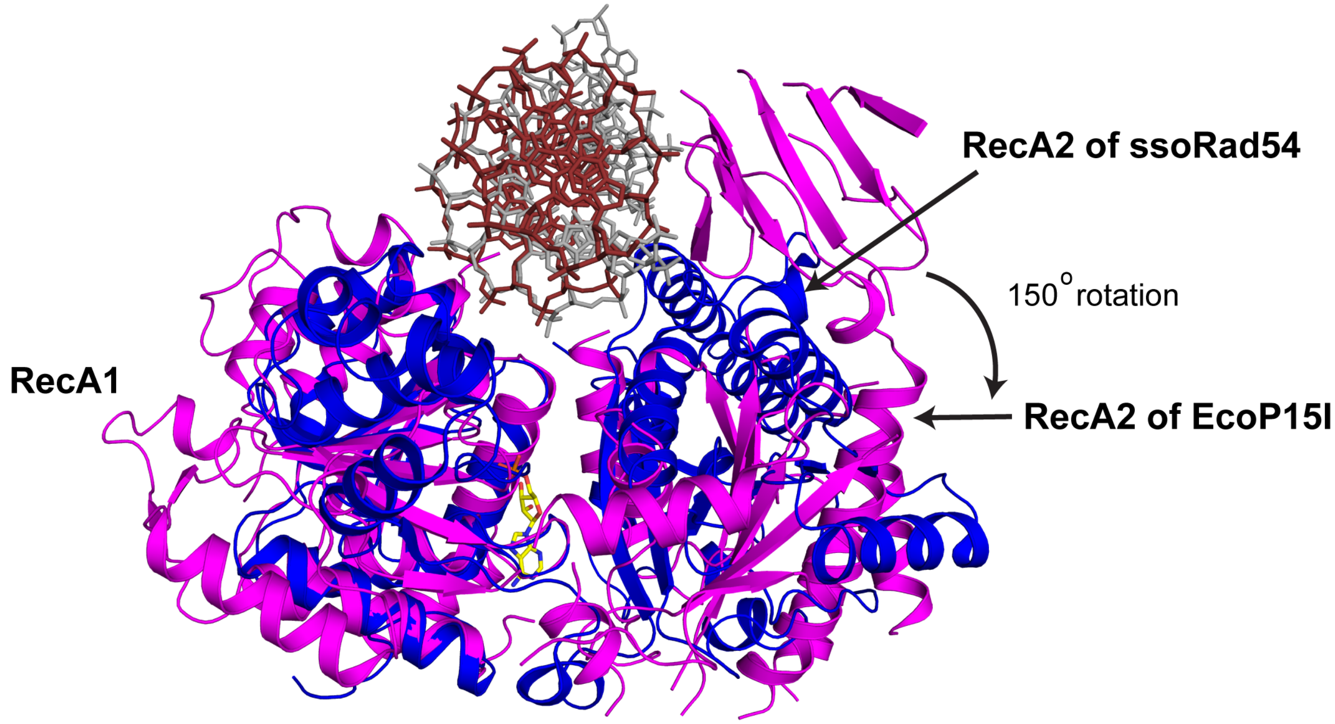


B

Superposition of walker A motifs of EcoP15I vs Vasa

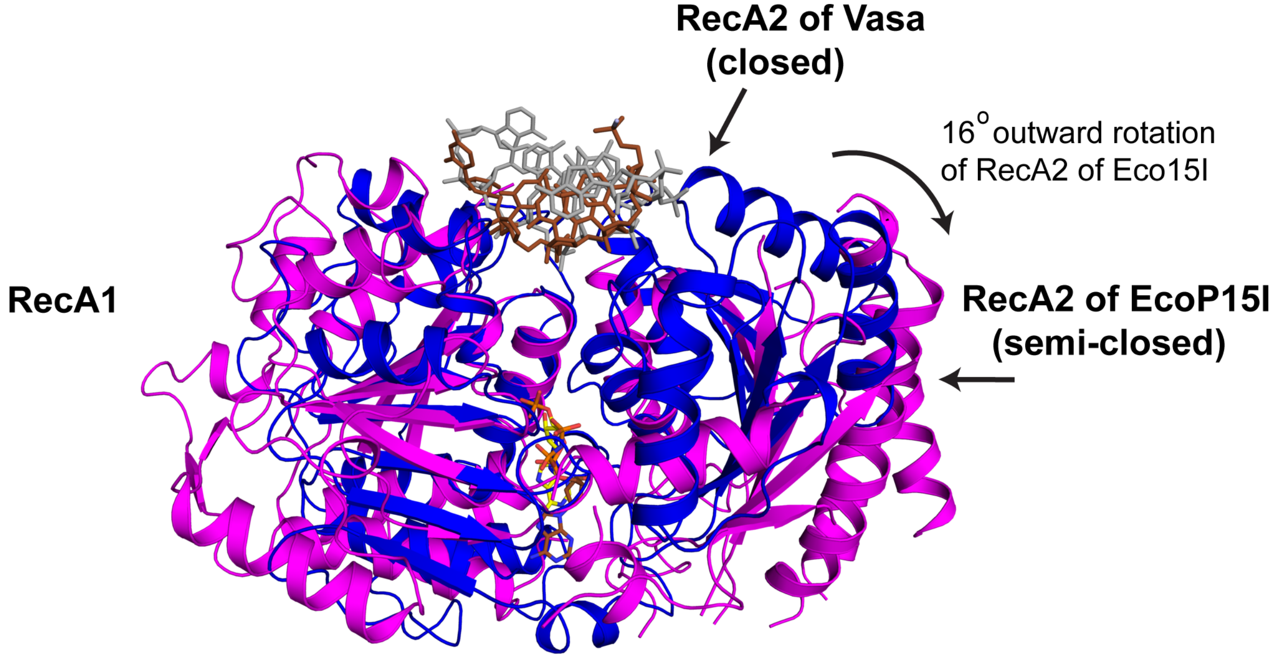

**Supplementary Figure 9.** ***Semi-closed*** **orientation of RecA domains in EcoP15I**

A) Walker A motif based superposition of helicase cores of EcoP15I (magenta) and ssoRAD54 (pdb id 1Z63, blue ribbons). B) Walker A motif based superposition of helicase cores of EcoP15I (magenta) and VASA (pdb id 2DB3, blue ribbons). The RecA1 and the translocating strand of EcoP15I overlay well on to the corresponding modules in both Vasa and ssoRAD54. The RecA2 in EcoP15I assumes a ~16° outward rotation compared to RecA2 of VASA and ~150° inward rotation compared to RecA2 of ssoRAD54 and may represent a *"semi-closed"* orientation of RecAs around substrate DNA. Pin domain is omitted for clarity in the lower panel. Gray sticks, EcoP15I's DNA; brown sticks, Vasa's RNA/RAD54's DNA and AMPPNP in VASA/AMP in EcoP15I; yellow sticks.

Supplementary figure 10

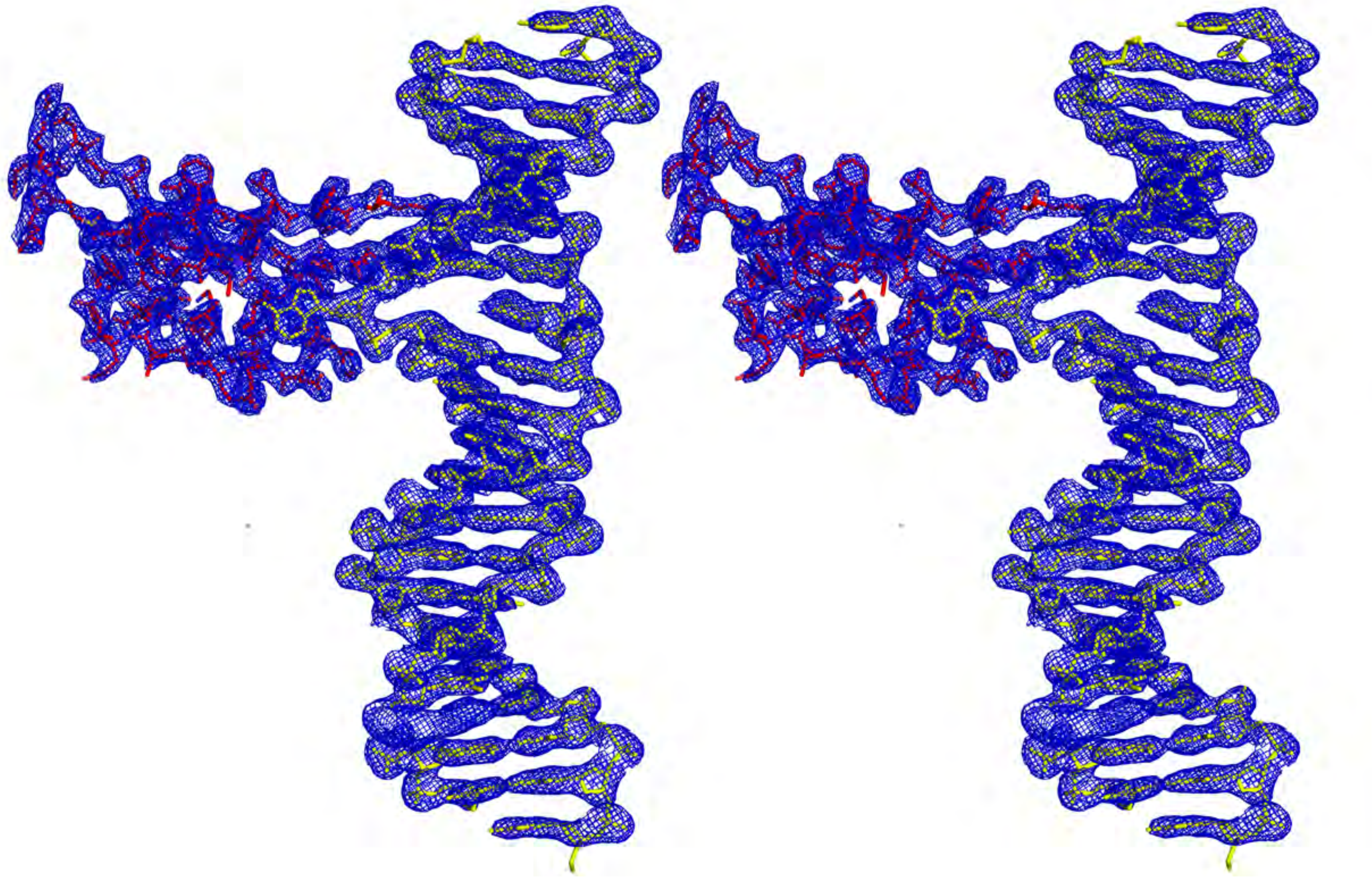

**Supplementary Figure 10. Stereo view of a section of electron density map**

A cross-eye stereo view of section of a 2Fo-Fc map (contoured at $\sigma = 1.7$) shows clear electron density of DNA and parts of ModB. The corresponding model to this part of the map is shown in stick mode.

## Supplementary Table 1. Summary of residues omitted or built as alanines

| | ModA | ModB | Res |
|---|---|---|---|
| Residues absent in model due to poor electron density | 1-12, 49-52, 140, 475, 525-529, 616 633-634 | 1, 8-9, 16, 31, 250, 372, 517-534, 568-569, 587, 588, 597-599, 615-616, 635-636 | 1-5, 154-162, 177, 288-292, 309-318, 324, 343-345, 393, 407-419, 437-443, 463-473, 522-526, 549-550, 578-582, 595-619, 634-666, 706-743, 775-779, 801-803, 811-970 |
| Residues built in model as alanines | 14, 16, 47, 91, 92, 130, 148, 155, 162, 166, 218, 278, 281, 294, 311, 312, 337, 338, 354, 362, 411, 416, 423, 458, 473, 474-477, 480, 481, 500, 517, 519, 521, 523, 524, 530, 534, 535, 570, 586, 595, 625, 629, 630, 635 | 2-4, 25, 27, 30, 32, 37-45, 48, 50, 53, 136, 138, 140, 148, 154, 259, 280, 281, 283, 286, 288, 311, 337, 358, 362-364, 373, 377, 386, 404, 415, 457, 461, 473, 474, 476, 535, 536, 569, 570, 590-596, 600, 601, 606, 619, 624, 625, 628-630, 632-634, 637 | 6, 8-10, 15, 27, 34, 38, 45, 4755, 58, 70, 76, 130, 141, 152, 153, 175, 179, 197, 233, 293-308, 319-346, 354, 356, 357, 360-362, 368, 369, 380, 387, 391, 392, 395, 420-436, 444-462, 474-477, 479, 482-83, 485, 492, 495, 501, 506, 513, 518-519, 521, 527, 530, 551, 561-564, 568-571, 573-576, 583-594, 620-633, 667-679, 686, 701-704,744, 746, 747, 751, 754, 755, 773-774, 780, 783, 785-787, 789-790, 794, 796-797, 799-808 |
| Outlier residues in Ramachandran plot | 91, 281, 457, 459 | 384, 504 | 509 |

**Supplementary Table 2** Closest structural homologues of functional subunits of EcoP15I*

PDBeFold (www.ebi.ac.uk/msd-srv/ssm/cgi-bin/ssmserver) results

| Query domain | Top 5 closest hits in pdb | **% SSE** (secondary structural elements matched in a pdb target & r.m.s.d.) |
|---|---|---|
| ***EcoP15I Mod*** | | |
| Mtase (aa 62-262, 390-516) | 1. MboIIA Mtase (pdb; 1g60)<br>2. TTHA0409 Mtase (pdb; 2zif)<br>3. PvuII Mtase (pdb; 1boo)<br>4. RsrI Mtase (pdb; 1nw7)<br>5. Putative Mtase (pdb 3lpm) | 59, 1.81<br>55, 1.74<br>55, 2.10<br>55, 1.93<br>36, 2.91 |
| CTD (aa 539-644) | 1. Response regulator from Colwellia psychrerythraea (pdb; 3eqz)<br>2. Receiver domain from Sinorhizobium meliloti (pdb; 1qkk)<br>3. Receiver domain of sensor histidine kinase CKI1 (pdb; 3mm4)<br>4. Response regulator CheY (pdb; 1d4z)<br>5. Hypothetical protein PFU-723267-001 (pdb; 1y81) | 75, 3.18<br>75, 3.43<br>75, 3.64<br>75, 3.10<br>75, 3.17 |
| ***EcoP15I Res*** | | |
| Helicase (RecA1+RecA2; aa 7-269, 366-594)) | 1. Vasa helicase from silk worm (pdb; 4d25)<br>2. Duck RIG-I helicase (pdb; 4a36)<br>3. Exon Junction complex/DEAD box (pdb; 2j0q)<br>4. Exon Junction complex with a trapped DEAD box (pdb; 2hyi)<br>5. Vasa helicase from drosophila (pdb; 2db3) | 47, 3.18<br>47, 2.83<br>47, 3.05<br>47, 2.94<br>47, 3.4 |
| Pin (aa 293-365) | 1. LIM domain from quail CRP (pdb; 1a7i)<br>2. LIM domain of LIM1 (pdb; 1x63)<br>3. Ribosomal protein (pdb; 4peb)<br>4. Male transporter NORM-NG (pdb; 4hum)<br>5. Ribosomal protein (pdb; 3j0y) | 100, 4.03<br>100, 4.62<br>75, 4.03<br>75, 4.21<br>75, 3.53 |
| Helical spacer (aa 620-810) | 1. Calprotectin S100A8 (pdb; 1xk4)<br>2. S100A13 (pdb; 2k8m)<br>3. Death domain (pdb; 2g7r)<br>4. Copper efflux regulator (pdb; 1q05)<br>5. S100C (pdb; 1qls) | 50, 3.63<br>50, 3.06<br>50, 3.62<br>50, 3.75<br>50, 3.81 |

## Supplementary References


1 Malone, T., Blumenthal, R. M. & Cheng, X. Structure-guided analysis reveals nine sequence motifs conserved among DNA amino-methyltransferases, and suggests a catalytic mechanism for these enzymes. *J Mol Biol* **253**, 618-632 (1995).

2 McClelland, S. E. & Szczelkun, M. D. in *Restriction Endonucleases* (ed A. Pingoud) 111-135 (Springer-Verlag, 2004).
